# Emerging therapies for autosomal dominant polycystic kidney disease with a focus on cAMP signaling

**DOI:** 10.3389/fmolb.2022.981963

**Published:** 2022-09-02

**Authors:** Xia Zhou, Vicente E. Torres

**Affiliations:** Mayo Clinic, Department of Nephrology, Rochester, MN, United States

**Keywords:** cAMP signaling, protein kinase A (PKA), ADPKD (autosomal dominant polycystic kidney disease), PKD, vassopressin, tolvaptan

## Abstract

Autosomal dominant polycystic kidney disease (ADPKD), with an estimated genetic prevalence between 1:400 and 1:1,000 individuals, is the third most common cause of end stage kidney disease after diabetes mellitus and hypertension. Over the last 3 decades there has been great progress in understanding its pathogenesis. This allows the stratification of therapeutic targets into four levels, gene mutation and polycystin disruption, proximal mechanisms directly caused by disruption of polycystin function, downstream regulatory and signaling pathways, and non-specific pathophysiologic processes shared by many other diseases. Dysfunction of the polycystins, encoded by the PKD genes, is closely associated with disruption of calcium and upregulation of cyclic AMP and protein kinase A (PKA) signaling, affecting most downstream regulatory, signaling, and pathophysiologic pathways altered in this disease. Interventions acting on G protein coupled receptors to inhibit of 3′,5′-cyclic adenosine monophosphate (cAMP) production have been effective in preclinical trials and have led to the first approved treatment for ADPKD. However, completely blocking cAMP mediated PKA activation is not feasible and PKA activation independently from cAMP can also occur in ADPKD. Therefore, targeting the cAMP/PKA/CREB pathway beyond cAMP production makes sense. Redundancy of mechanisms, numerous positive and negative feedback loops, and possibly counteracting effects may limit the effectiveness of targeting downstream pathways. Nevertheless, interventions targeting important regulatory, signaling and pathophysiologic pathways downstream from cAMP/PKA activation may provide additive or synergistic value and build on a strategy that has already had success. The purpose of this manuscript is to review the role of cAMP and PKA signaling and their multiple downstream pathways as potential targets for emergent therapies for ADPKD.

## Introduction

Autosomal dominant polycystic kidney disease (ADPKD), the most common inherited renal cystic disease, has an estimated genetic prevalence between 1:400 and 1:1,000 individuals and is responsible for 5%–10% of kidney failure world-wide (Bergmann et al., 2018). It is characterized by development of numerous tubular diverticula which become cysts after detaching. Unrelenting cyst growth causes kidney enlargement and functional decline, associated with hypertension, cyst hemorrhage, gross hematuria, nephrolithiasis, cyst infection, pain, and reduced quality of life. The total annual costs attributed to ADPKD in the United States were estimated to be $7.3 to $9.6 billion in 2018 (Cloutier et al., 2020). Direct healthcare costs accounted for $5.7 billion (78.6%), mostly driven by renal replacement therapy ($3.2 billion; 43.3%). Indirect costs accounted for $1.4 billion (19.7%), mostly driven by productivity loss due to unemployment ($784 million; 10.7%) and reduced productivity at work ($390 million; 5.3%).

## Progress in the understanding of polycystic kidney disease

**FIGURE 1:**
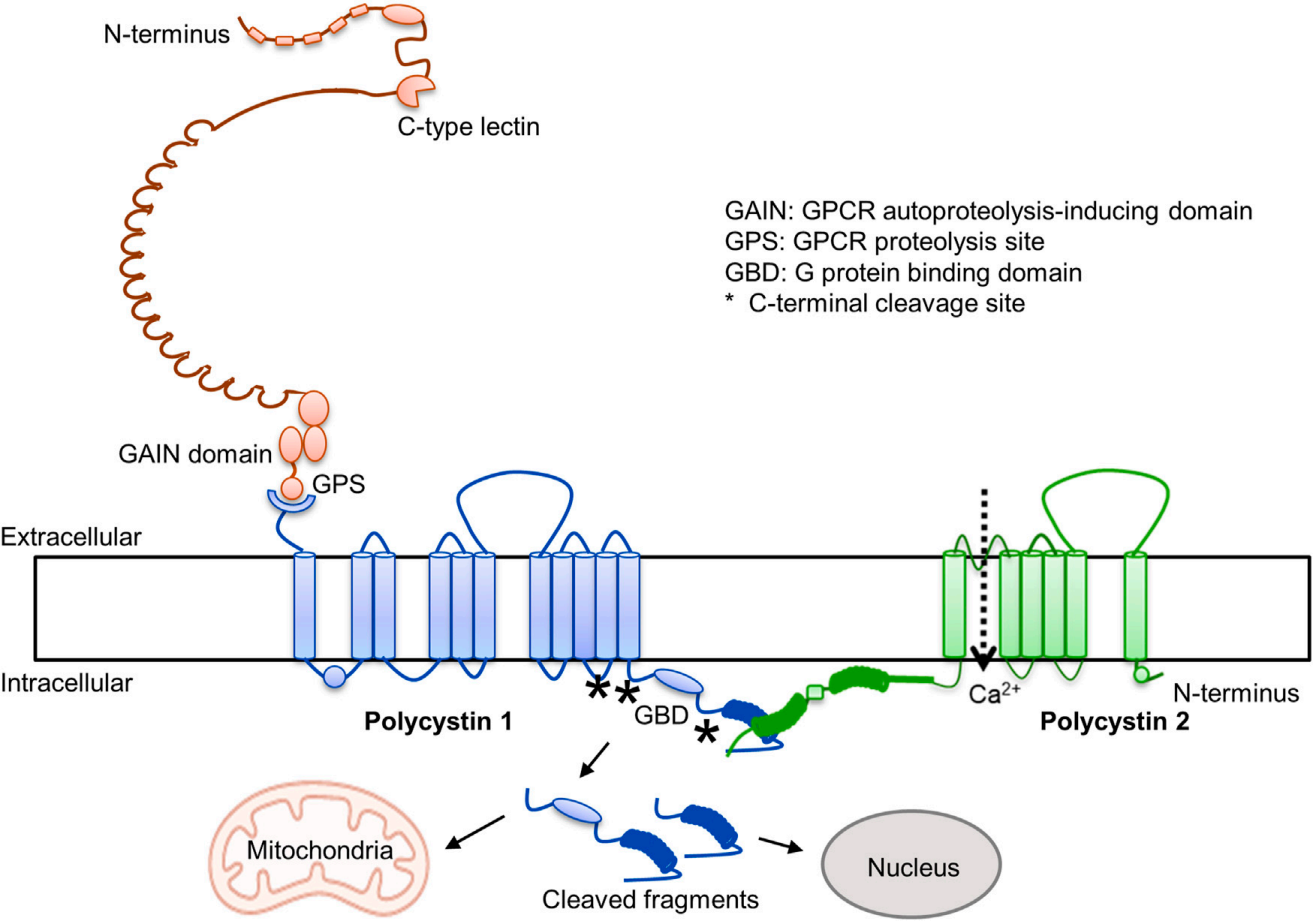
Polycystin 1 and polycystin 2.

Over the last 3 decades there has been great progress in the understanding of ADPKD (1). It is caused by mutations in *PKD1* and *PKD2* and disruption of the encoded proteins (polycystin-1 and polycystin-2). Polycystin 1 is an adhesion type G protein coupled receptor, characterized by the presence of many adhesive extracellular domains, a G protein autoproteolytic site, and an intracellular G protein binding domain (Figure 1). The cleavage at the autoproteolytic site generates a N-terminal fragment and a stalk that binds to extracellular loops of the C-terminal domain and regulates Gα- and Gβγ-protein signaling, possibly inhibiting cAMP production (Maser and Calvet, 2020). Polycystin-2 is a transient receptor potential protein and interacts with polycystin-1 in a three to one ratio to form heterotetramer channels permeable to calcium. It has been proposed that the C-lectin in the N-terminal fragment of PC1 interacts with the top domain of PC2 to activate the channel (Ha et al., 2020). G-protein and calcium signaling reciprocally interact, G proteins regulating intracellular calcium dynamics and intracellular calcium regulating cAMP synthesis and degradation (Sussman et al., 2020). In addition, the C-terminal tail of polycystin-1 undergoes regulated cleavage with formation of cleaved fragments, containing mitochondrial and nuclear targeting sites, that translocate to nuclei and mitochondria may also regulate downstream signaling (Padovano et al., 2020). Dysregulation of G protein and intracellular calcium signaling, and possibly of signaling regulated by polycystin-1 C-terminal tail cleaved fragments, alter multiple pathways and pathophysiologic processes and promote cystogenesis.

## Stratification of therapeutic targets

**FIGURE 2:**
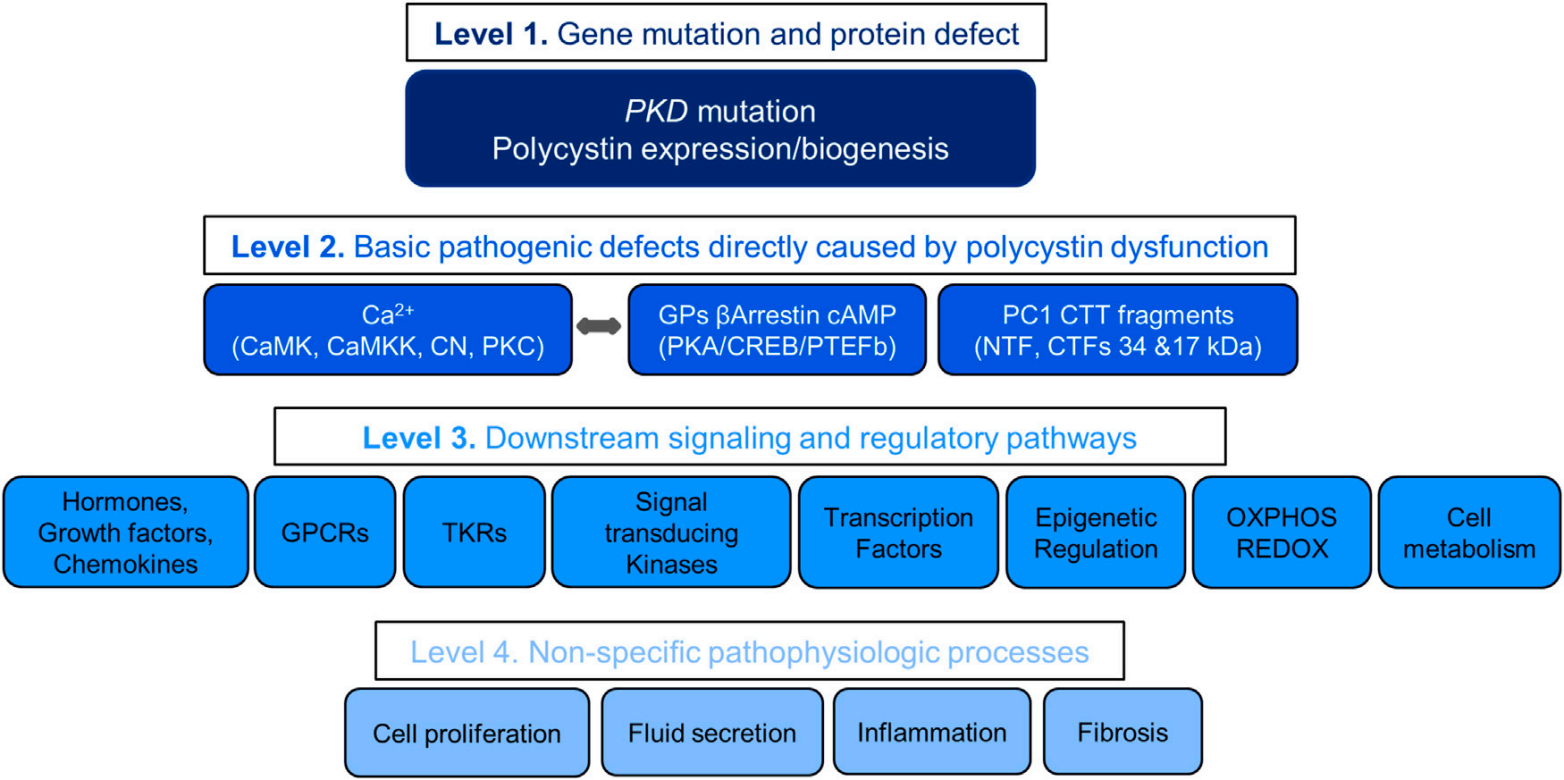
Levels of Therapeutic targets.

Understanding the pathogenesis of ADPKD allows the stratification of therapeutic targets into four levels with decreasing likelihood of therapeutic efficacy (Figure 2). The first includes the gene mutations and protein disruptions. The second, the basic pathogenic mechanisms directly caused by the disruption of polycystin function. The third, downstream signaling and regulatory pathways. The fourth, non-specific pathophysiologic processes shared by many other renal and non-renal diseases. Redundancy of mechanisms, numerous positive and negative feedback loops, and possibly downstream counteracting effects may account for the limited, although statistically significant effectiveness of many treatments in preclinical studies. Few of over 150 compounds effective in rodent models of PKD have been tested in clinical trials, mostly with negative results. Failure in clinical trials has in part been due to toxicity of the compounds (limiting the dosing feasible in human trials) and possibly development of somatic mutations or reprogramming of the epigenome, transcriptome or kinome of the cystic cells.

## Central role of cAMP in the pathogenesis of ADPKD

Overwhelming evidence supports the central role of cAMP in ADPKD, possibly by promoting cyst initiation and most definitely by stimulating proliferation of the cystic epithelium and fluid secretion into the cysts, thus promoting cyst growth.

### Cyst initiation

Enhanced cAMP and protein kinase A (PKA) signaling disrupts tubulogenesis. Epithelial tubulogenesis requires canonical Wnt/β-catenin signaling at early inductive stages and noncanonical Wnt/planar cell polarity signaling later. PKA is known to enhance Wnt/β-catenin signaling through phosphorylation of glycogen synthase kinase 3b (stabilizing β-catenin) and phosphorylation of β-catenin (promoting its transcriptional activity) (Li et al., 2000; Taurin et al., 2006). Sustained PKA-dependent canonical Wnt signaling blocks a post-epithelialization morphogenetic step (conversion of the renal vesicle to the S-shaped body) in spinal cord-induced metanephric mesenchyme, resulting in disorganized epithelial clusters and large dilations (Gallegos et al., 2012). Overexpression of constitutively active PKA catalytic subunits can also act as a negative regulator of planar cell polarity signaling and block convergent extension during Xenopus gastrulation (Song et al., 2003). Deletion of polycystin-1 increased cAMP and switched tubule formation by principal-like MDCK cells to cyst formation, and pharmacological elevation of cAMP in polycystin-1-competent cells caused cyst formation, impaired plasticity, nondirectional migration, and mis-orientation strongly resembling the phenotype of polycystin-1-deficient cells (Scholz et al., 2022). Mis-orientation of developing tubule cells in metanephric kidneys upon loss of polycystin-1 was also phenocopied by pharmacological increase of cAMP in wildtype kidneys. These observations suggest that cAMP triggers the initiation of cyst formation and not only promotes cyst enlargement in ADPKD.

### Proliferation of the cystic epithelium

cAMP stimulates the proliferation of ADPKD cells but inhibits the proliferation of normal human kidney cells (Yamaguchi et al., 2003; Yamaguchi et al., 2004; Yamaguchi et al., 2006). The proliferative effect on ADPKD cells is due to aberrant crosstalk between intracellular calcium and cAMP. Intracellular calcium is reduced in the cystic epithelium and cAMP is increased. Restoration of intracellular calcium with a channel activator or low levels of a calcium ionophore converts the proliferative to an antiproliferative effect. In contrast, decreasing the intracellular calcium converts the antiproliferative to a proliferative response in the normal epithelium. How intracellular calcium determines whether cell proliferation is stimulated or inhibited by cAMP is not well understood. It has been proposed that a reduction in intracellular calcium de-represses BRAF causing cAMP activation of MEK-ERK and increased cell proliferation (Yamaguchi et al., 2004). Collecting duct specific expression of constitutively active BRAF induces cystogenesis in wildtype, and accelerates cyst growth, inflammation, and fibrosis in slowly progressive models of PKD (Ramalingam et al., 2021).

### Fluid secretion into the cysts

Fluid secretion into the cysts is driven by cAMP-dependent chloride secretion, involving the basolateral Na-K-Cl cotransporter (NKCC1) and the apical cystic fibrosis transmembrane conductance regulator (CFTR) chloride channel (Sullivan et al., 1998; Grantham, 2003). The stimulation of chloride driven fluid secretion by cAMP is common to many secretory epithelia including normal collecting ducts and kidney tubules. Extracellular chloride entry by the basolateral NKCC1 (Kim et al., 1999; Gonin et al., 2001; Ortiz, 2006) raises its intracellular chloride concentration above the electrochemical gradient for chloride efflux. In the presence of cAMP agonists, CFTR channels are activated by PKA dependent phosphorylation and chloride flows into the cysts. The transepithelial transport establishes an electrical gradient for sodium transport through the paracellular pathway.

## Importance of vasopressin

The evolution of vasopressin-related peptides dates back more than 700 million years, prior to the appearance of kidneys and pituitary gland (Juul, 2012). The evolutionary emergence of vasopressin, arginine vasopressin receptor 2 (Avpr2), and urine-concentrating mechanisms paralleled the development of loops of Henle and renal medulla and of nephron heterogeneity (short- and long-looped nephrons) in mammals. Lack of nephron heterogeneity in homozygous Brattelboro rats lacking vasopressin and induction of anatomic changes, i.e., hypertrophy and elongation of the thick ascending limb of Henle’s loop and the inner stripe of the outer medulla by the administration of the vasopressin V2 receptor (V2R) agonist 1-deamino-8-d-arginine vasopressin suggest a mechanistic coupling of these evolutionary changes (Trinh-Trang-Tan et al., 1987).

**FIGURE 3:**
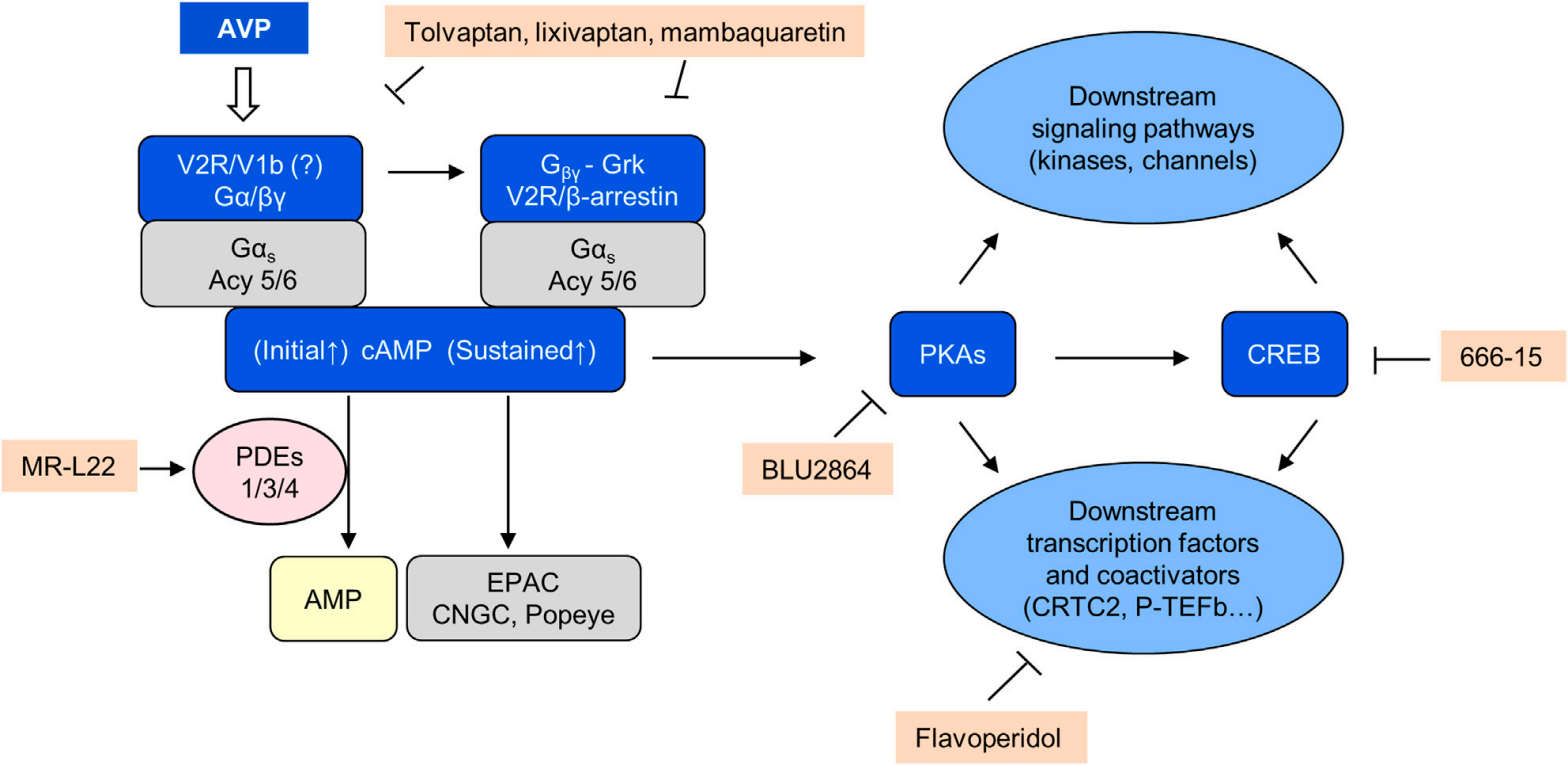
AVP/cAMP/PKA signaling.

Prolonged and sustained elevation of circulating vasopressin by osmotic stimulation or through continuous infusion of vasopressin for at least 3 days induces a proliferative response in cells expressing V2Rs (thick ascending limb of Henle and collecting duct) that was blocked by V2R but not by V1a or V1b receptor antagonists (Naito et al., 2001; Alonso et al., 2009). These observations suggest that prolonged V2R stimulation can induce to a cAMP-dependent proliferative phenotype in distal tubular and collecting duct cells (Figure 3).

Vasopressin acting on V2Rs is the main agonist of adenylyl cyclase in freshly dissociated collecting ducts (Yasuda and Jeffries, 1998). The kidneys are continuously exposed to the tonic action of vasopressin to avoid dehydration. This exposure is further enhanced in PKD owing to a concentrating defect distal to cAMP generation and PKA activation (Gattone et al., 1999; Seeman et al., 2004). Levels of cAMP are increased in cystic tissues (Yamaguchi et al., 1997). These observations together with the importance of cAMP for cyst initiation and progression provided a strong rationale for strategies to inhibit its production (Gattone et al., 1999; Gattone et al., 2003). Moreover, the almost exclusive localization of V2Rs on collecting ducts, connecting tubules, and thick ascending limbs of Henle (Mutig et al., 2007), the main sites of cystogenesis (Verani and Silva, 1988) predicted few off-target toxicities.

## Preclinical trials targeting the vasopressin V2R and other GPCRs

Cyst development was markedly inhibited in PCK rats lacking circulating vasopressin (generated by crosses of PCK and Brattleboro rats), an effect reversed by the administration of the V2R agonist 1-deamino-8-d-arginine vasopressin (Wang et al., 2008). Suppression of vasopressin by high water intake sufficient to achieve a 3.5-fold increase in urine output attenuated the progression of PKD in PCK and LPK rats but not in *Pkd1* ^RC/RC^ mice (Nagao et al., 2006; Hopp et al., 2015b; Sagar et al., 2019). Vasopressin V2 receptor antagonists (mozavaptan, tolvaptan, lixivaptan and mambaquaretin-1) ameliorated PKD in multiple orthologous (*Pkd2* ^WS25/−^, *Pkd1* ^RC/RC^ mice; PCK rat) and non-orthologous (pcy mouse) rodent models (Gattone et al., 2003; Torres et al., 2004; Wang et al., 2005; Zittema et al., 2016; Ciolek et al., 2017; Di Mise et al., 2019; Wang et al., 2019; Arroyo et al., 2021). Sustained suppression of vasopressin V2R was critical for the protective effect (Aihara et al., 2014).

Somatostatin, a hormone secreted by cells of the nervous system, gastrointestinal tract, and pancreatic islets, acts on five GPCRs (SSTR1 to 5) coupled to Gi proteins, inhibits cAMP generation in MDCK cells and rat collecting ducts, and antagonizes vasopressin effects in the toad urinary bladder and dog collecting ducts (Winkler et al., 1982; Friedlander and Amiel, 1986; Parnell et al., 2022). It also inhibits the secretion of several hormones and growth factors such as growth hormone, IGFI and vascular endothelial growth factor that promote cyst growth. Consistent with these observations, somatostatin analogs ameliorated PKD and polycystic liver disease in several orthologous models (*Pkd1* ^RC/RC^ and *Pkd2* ^WS25/-^ mice, and PCK rats) (Masyuk et al., 2007; Masyuk et al., 2013). Furthermore, tolvaptan and pasireotide, a synthetic analogue of somatostatin, had an additive effect inhibiting the development of the renal cystic disease in *Pkd1* ^RC/RC^ mice as characterized by decreased kidney weight to body weight ratio, cystic and fibrotic volume, and cAMP Level (Hopp et al., 2015a).

Other GPCRs coupled to Gαs proteins also may contribute to the development of PKD and their blockade has been protective in animal models. The β3-adrenergic receptor (β3-AR) is expressed the loops of Henle and cortical collecting ducts and is found at high levels in murine and human polycystic kidneys (Procino et al., 2016). Selective activation of β3-AR increases cAMP levels in isolated mouse renal tubules and activates key proteins involved in transepithelial water and solute movement. β3-AR blockade with a selective antagonist (SR59230A) decreases cAMP levels and ameliorates the cystic phenotype (Schena et al., 2021). TGR5 is overexpressed in cystic kidneys and livers and its activation by bile acids increases cAMP and proliferation. The TGR5 agonist, oleanolic acid, worsened renal and hepatic cystogenesis in PCK rats, whereas genetic elimination of *Tgr5* ameliorated the fibropolycystic liver disease of *Pkhd1* knockout mice (Masyuk et al., 2017). PGE_2_ acting on EP2 or EP4 receptors promote cystogenesis in 3D-culture which is abolished by selective EP2 and EP4 antagonists (Lannoy et al., 2020). Unexpectedly, selective EP2 (PF-04418948) or EP4 (ONO-AE3-208) antagonists aggravated the cystic disease in two (hypomorphic and inducible) *Pkd1* mouse models, possibly related to unexpected pro-inflammatory effects (Lannoy et al., 2020).

## Clinical trials indirectly or directly targeting GPCRs

Only 3 drugs are currently used in clinical practice to specifically treat PKD or polycystic liver disease (all targeting cAMP signaling), and only one has been approved by the FDA, EMA, and other regulatory agencies (tolvaptan).

Two large randomized clinical trials of the V2 receptor antagonist tolvaptan, TEMPO 3:4 in patients with CKD 1 and 2 and REPRISE in patients with an eGFR between 25 and 65, led to its approval for patients with rapidly progressive ADPKD (Torres et al., 2012; Torres et al., 2017). TEMPO 3:4 was enriched for patients with rapidly progressive disease and showed a reduction in kidney growth of 49% over 3 years. Both trials showed a reduction in the rate of decline of GFR. TEMPO 3:4 showed a reduction in kidney pain.

TEMPO 4:4, an open label extension of TEMPO 3:4, showed that the benefit of 3:4 was sustained and a single center longer follow-up study showed that it was cumulative over time (Torres et al., 2018a; Edwards et al., 2018). Two additional studies have been reported in the last year. An open label extension of REPRISE showed effectiveness of tolvaptan at very advanced stages of CKD (Torres et al., 2021). A pediatric randomized double-blind trial showed that the rates of kidney growth and eGFR decline were substantially less in the tolvaptan than in the placebo group, without reaching statistical significance, possibly due to the small size of the groups, heterogeneity of study patients, and short duration of follow-up (Mekahli et al., 2021).

Recently, the results of a randomized clinical trial of high prescribed water intake show no significant effect on the rate of kidney growth or decline of kidney function, but was inconclusive because the target 24 h urine osmolality was achieve in only half of the patients without any difference in the plasma copeptin levels between the groups (Rangan et al., 2022).

Three large trials have assessed the efficacy of somatostatin analogs for PKD, Aladin 1 and DIPAK mainly in patients with CKD 3 and Aladin 2 mainly in patients with CKD 3B and 4 (Caroli et al., 2013; Meijer et al., 2018; Perico et al., 2019). All showed a significant treatment effect slowing the rate of kidney growth. Two studies showed no significant effect on the rate of eGFR decline, one study showed a non-significant slowing trend after the first year, and another showed a reduced risk for doubling the serum creatinine or reaching ESKD. All the studies, plus additional trials on PLD, showed a significant treatment effect on liver growth (van Keimpema et al., 2009; Hogan et al., 2010; Hogan et al., 2012; Hogan et al., 2020).

Given the central and proximal role of cAMP, the efficacy of tolvaptan and somatostatin analogs in preclinical and clinical trials, and the inability of these drugs to completely block renal cAMP production, targeting other links in the cAMP signaling pathway seems logical. Currently the mechanisms by which cAMP promotes the development and progression of ADPKD are not completely understood and opportunities for targeting cAMP signaling in ADPKD have not been exhausted and remain feasible. Furthermore, binding of vasopressin or somatostatin to their GPCRs may also affect signaling pathways independent from cAMP.

## Complexity of GPCR signaling

GPCRs consist of an extracellular amino-terminal domain, seven transmembrane spanning α-helices, and an intracellular carboxyl tail (Katritch et al., 2013; Zhang et al., 2015; Hilger et al., 2018; Chan et al., 2019; Sussman et al., 2020). Ligand binding to GPCRs rearranges its transmembrane helices and facilitates coupling to heterotrimeric G-proteins. These are composed of Gα subunits (Gαs, Gαi/o, Gαq/11 or Gα12/13) and Gβγ-dimers. Ligand binding promotes exchange of GDP bound to the Gα subunit for GTP. GTP-bound Gα dissociates from the receptor and from Gβγ, and Gα and Gβγ separately mediate downstream G protein signaling. Gαs and Gαi bind directly to adenylyl cyclase increasing or decreasing cAMP production. Gαq and Gα11 activate phospholipase C. Phospholipase C hydrolyses membrane lipid phosphatidylinositol 4,5-bisphosphate to inositol 1,4,5-trisphosphate and diacylglycerol, each initiating a signal transduction cascade. Gα12 and Gα13 activate Rho. Gβγ subunits interact with many effector proteins and have many biological functions. GPCR-bound Gβγ recruits GPCR kinases (GRK) that phosphorylate specific serine and threonine residues of agonist-activated GPCRs within its carboxy terminal domain and promote the binding of β-arrestins.

## Relevance of β-arrestins as negative and positive regulators of vasopressin and G protein signaling

The canonical β-arrestin function is the negative regulation of G protein signaling by several mechanisms (Jean-Charles et al., 2017; Peterson and Luttrell, 2017). β-arrestins bind to phosphorylated residues in the GPCR C-terminal tail and in the transmembrane core (third intracellular loop). Since the binding site in the GPCR core overlaps with the G-protein-binding site, β-arrestin recruitment hinders further G protein activation. β-arrestins couple GPCRs to clathrin and adaptor protein-2, which mediate endocytosis of GPCRs and desensitization. Furthermore, β-arrestins may limit GPCR signaling by acting as a scaffold for cyclic nucleotide phosphodiesterases and diacylglycerol kinases.

More recent evidence suggests that some internalized GPCR/β-arrestin complexes result in sustained activation of adenylyl-cyclase and/or β-arrestin dependent activation of Src, extracellular signal regulated kinase (ERK1/2), c-Jun-N-terminal kinase (JNK), and p38 MAPK (Shenoy et al., 2006; Pakharukova et al., 2020; Kim et al., 2022). The extent of GRK-mediated phosphorylation determines the stability of GPCR/β-arrestin complexes. GPCRs with few phosphorylation sites (e.g., β2 adrenergic receptor) interact with β-arrestin in endosomes with low affinity and rapidly recycle back to the plasma membrane. GPCRs with many phosphorylation sites (e.g., vasopressin V2 receptor or V2R) exhibit sustained high-affinity interactions with β-arrestin in endosomes and slowly recycle or traffic to lysosomes for degradation (Luttrell et al., 2001; Beautrait et al., 2017; Jean-Charles et al., 2017; Thomsen et al., 2018; Baidya et al., 2020). When β-arrestins bind only through the C tail to promote internalization, the receptor core region is exposed and the GPCR can interact simultaneously with both G proteins and β-arrestins and induce G-protein and/or β-arrestin signaling. Thus, β-arrestins can act as negative or positive regulators of G protein signaling at the plasma membrane or endosomes, respectively. Once activated GPCRs can selectively promote the activation of G-protein or β-arrestin signalling, a phenomenon known as functional selectivity or ligand bias.

The affinity of the agonist for the receptor and the stability of the agonist/GPCR interaction also affects the balance between plasma membrane and endosomal signaling. Both vasopressin and oxytocin bind to the V2R. Vasopressin binds tightly to the V2R, which results in prolonged internalization and endosomal G protein signaling, whereas oxytocin binds to V2R with lower affinity and dissociates from the receptor soon after internalization, which results in predominant plasma membrane G protein signaling (Thomsen et al., 2018). While vasopressin promotes cAMP generation at the plasma membrane and sustained cAMP accumulation at endosomes after β-arrestin mediated receptor internalization (Figure 3), oxytocin only induces a transient generation of cAMP at the plasma membrane and does not cause β-arrestin binding or V2R internalization. Consistent with these effects, vasopressin has strong antidiuretic and antinatriuretic effects as opposed to oxytocin which has weak effects only measurable in the absence of natural vasopressin. The selective β-arrestin/β2-adaptin inhibitor barbadin prevents the vasopressin-promoted endocytosis of the V2 receptor, cAMP accumulation, and ERK1/2 activation (Beautrait et al., 2017).

Several observations suggest that V2R/β-arrestin signaling may be important in PKD. First, the effect of vaptans on PKD is moderate compared to that of genetic elimination of circulating vasopressin (Wang et al., 2008). This may be because vaptans inhibit the V2R-dependent G-protein signaling but exhibit a partial agonist activity on β-arrestin recruitment and MAPK activation. Second, although secretin, like vasopressin, activates adenylyl cyclase in the outer medulla and decreases urine output in wild-type and Brattleboro rats, genetic elimination of the secretin receptor and administration of exogenous secretin to PCK or PCK/Brattleboro rats or to *Pkd2* ^-/WS25^ mice did not significantly affect PKD (Wang et al., 2012). This may be because the internalization of the secretin receptor is unaffected by GRK mediated phosphorylation or by the expression of dominant negative β-arrestin (Walker et al., 1999). Recruitment of β-arrestins to GPCRs after agonist activation is to large extent dependent on β/γ signaling. Third, inhibition of β/γ signaling using gallein or knocking out the G-protein β subunit, likely inhibiting β-arrestin signaling, corrected the phenotype of Xenopus *pkd1* morphants and inhibited cystogenesis of *Pkd1* ^−/−^ cells in 3D culture (Zhang et al., 2018). Finally, expressions of β-arrestin 1 and/or 2 are increased in murine polycystic and human ADPKD kidneys (Xu et al., 2018).

At present, the role of β-arrestins in the pathogenesis of PKD remains unexplored. Interestingly, vaptans block cAMP generation but moderately stimulate β-arrestin signaling, whereas the mambaquaretin-1, a peptide from green mamba venom, inhibits both, cAMP generation and β-arrestin signaling (Ciolek et al., 2017). A study comparing the effectiveness of vaptans and mambaquaretin-1 has not been done.

## Adenylyl cyclases as therapeutic targets in ADPKD

cAMP is produced by nine membrane-bound adenylyl cyclase (AC) isoforms (ACs1–9) activated by GαsPCRs or one soluble AC (AC10) (Dessauer et al., 2017; Bassler et al., 2018) activated by bicarbonate and calcium. ACs 6 and 5 mRNA and protein levels are increased, whereas AC3 levels are decreased in ADPKD compared with NHK kidneys and cells. Calcium directly inhibits AC5 and AC6, whereas calcium/calmodulin activates and CaMKII inhibits AC3. All three contribute to the synthesis of cAMP in response to vasopressin (Strait et al., 2010). AC6 knockout mice have a concentrating defect, whereas AC3 knockout mice are normal, casting doubt on the relevance of AC3 in the regulation of water permeability of the collecting duct (Roos et al., 2012; Kittikulsuth et al., 2014). The cAMP increase in response to vasopressin *in vitro* is blunted in ADPKD compared to NHK cells and is mediated by AC3 (Pinto et al., 2012). AC3 inhibition does not affect AVP-induced cAMP production in NHK cells. The different response is thought to be due to reduced intracellular calcium, since it is reproduced in NHK cells treated with a calcium chelator or a calcium channel blocker. The relevance of these observations to *in vivo* conditions is uncertain. Mice with a collecting duct-specific double knockout of PC1 and AC6 have markedly decreased kidney and renal cyst volumes, improved renal function, reduced activation of the B-Raf/ERK/MEK pathway, and increased survival compared to mice with collecting duct-specific knockout of PC1 alone (Rees et al., 2014). In contrast, mice with a conditional *PC2*/*AC6* double knockout have no decrease in liver cyst volume compared to *Pkd2* knockout alone, whereas AC5 siRNA and/or inhibitors inhibit cAMP production and pERK1/2 expression by PC2 deficient cholangiocytes, growth of PC2 deficient biliary organoids, and liver cystic area and cell proliferation in conditional *Pkd2* knockout mice. A third study showed that knockdown of either AC5 or AC6 attenuated the increase in cAMP levels in PC2 deficient renal epithelial cells (Wang et al., 2018) and that *AC5*/*Pkd2* double mutant mice had less kidney enlargement, lower cyst index, and improved kidney function compared to *Pkd2* mutant mice.

Adenylyl cyclase inhibition has not been directly tested in animal models of PKD. Nevertheless, it might have contributed to the effect observed with some drugs directed against other targets. Metformin inhibits complex 1 of the mitochondrial respiratory chain (El-Mir et al., 2000), lowering ATP production and increasing AMP (Owen et al., 2000). AMP in turn inhibits adenylyl cyclase and activates AMPK. The inhibition of adenylyl cyclase (Miller et al., 2013) and activation of phosphodiesterase PDE4B by AMPK-mediated phosphorylation (Johanns et al., 2016) have been found to lower cAMP in response to metformin. Statins may lower cAMP through the downregulation of Gα s protein (Kou et al., 2012). Demeclocycline, and to a lesser extent doxycycline and other tetracycline antibiotics, have been shown to reduce urinary concentrating ability and have been used to treat the syndrome of inappropriate antidiuretic hormone in humans (Kortenoeven et al., 2013). In part, this is due to their capacity to reduce the expression of AC5, AC6 and AC3 without affecting their protein stability. By this mechanism, they could have a beneficial effect on PKD. Treatment with doxycycline aimed at inhibiting metalloproteinases indeed inhibited epithelial cell proliferation and cystic disease progression in PCK rats (Liu B. et al., 2012). However, the nephrotoxicity of these drugs when used at high doses may limit their potential for the treatment of PKD; for example, doxycycline at a high dose was found to aggravate cyst growth and fibrosis in pcy mice (Osten et al., 2009).

## cAMP phosphodiesterases as therapeutic targets in ADPKD

Phosphodiesterases control cAMP accumulation by promoting cAMP degradation. PDE 1, 3 or 4 control cAMP pools that regulate cell proliferation, fluid secretion, and cystogenesis in human cyst-derived epithelial cells *in vitro*, zebrafish and mouse models (Wang et al., 2010; Pinto et al., 2016; Sussman et al., 2016; Ye et al., 2016; Wang et al., 2017). Therefore, it seems likely that PDE activators would have a beneficial effect. Unfortunately, development of PDE activators has almost been non-existent, in contrast with great interest on the development and clinical applications of PDE inhibitors.

Long forms of PDE4 are activated by PKA phosphorylation as a feedback mechanism to terminate physiological cAMP mediated signaling. Small molecule compounds that mimic the effect of PKA phosphorylation and inhibit forskolin induced accumulation of cAMP have been developed by Mironid (Omar et al., 2019). These compounds inhibit *in vitro* cystogenesis of IMCD3 cells in matrigel and have been shown to lower kidney cAMP and ameliorate PKD in *Pkd1* ^RC/RC^ mice to a degree comparable to tolvaptan with less effect on urine output (Henderson et al., 2020).

It has been proposed that reduced cytosolic calcium is responsible for inhibition of the calcium dependent PDE1 and increased cAMP levels in cystic tissues and for the proliferative response of these cells to cAMP. Both provide a rationale for treatments increasing intracellular calcium. TRPV4 activators significantly alleviated cystic disease development in PCK rats (Gradilone et al., 2010; Zaika et al., 2013). Administration of triptolide, a drug that has been proposed to activate the PC2 channel by a poorly understood mechanism, has an inhibitory effect on the development of PKD in *Pkd1* ^−/−^ embryos and in kidney specific *Pkd1* knockout mice and in Han:SPRD rats (Leuenroth et al., 2007; Leuenroth et al., 2008; Leuenroth et al., 2010; Jing et al., 2018). Activation of the calcium-sensing receptor has been shown to increase intracellular calcium and decrease cAMP and mTOR in *PKD1* deficient cells (Di Mise et al., 2018). Calcimimetic agents ameliorated PKD in Cy/+ Han:SPRD rats and pcy mice, but not (except for reduced fibrosis) in PCK rats and *Pkd2* ^WS25/−^ mice, possibly because the potential beneficial effect of the drug was offset by marked hypocalcemia (Gattone et al., 2009; Wang et al., 2009; Chen et al., 2011). More recently, a lower dose of calcimimetic R-568 decreased intracellular cAMP level by the activation calcium-sensing receptor, reduced cyst progression in PCK rats and *Pkd1* ^RC/RC^ mice, and had an additive effect when given in combination with the V2 receptor antagonist lixivaptan (Di Mise et al., 2021). On the other hand, interventions that lower intracellular calcium aggravate the development of PKD. The administration of verapamil aggravated cyst development in Cy/+ Han:SPRD rats (Nagao et al., 2008). Treatment of zebrafish with CaV1.2 morpholinos induced pronephric duct cysts (Jin et al., 2014). Lentiviral transfection of CaV1.2 shRNA aggravates the cystic phenotype in *Pkd1* ^+/−^ mice (Jin et al., 2014).

## PKA as a direct therapeutic target

Direct inhibition of PKA is appealing because PKA can also be activated in PKD independently from cAMP by mechanisms associated with oxidant conditions, NFkB activation and TGFb activation (Zhong et al., 1997; Dulin et al., 2001; Zhang L. et al., 2004; Brennan et al., 2006; Yang H. et al., 2008; Lignitto et al., 2011; Zhou et al., 2012; Lignitto et al., 2013; Liu et al., 2014; Rinaldi et al., 2015; Hama et al., 2017; Sun et al., 2017). PKA is a tetramer with two catalytic subunits that remain inactive while bound to two regulatory subunits (Skalhegg and Tasken, 2000; Tasken and Aandahl, 2004; Yu et al., 2012; Baro Graf et al., 2020). When cAMP binds to the regulatory subunits the catalytic subunits are released and become active. Knocking out *Prkar1a* (coding for RIα, the most ubiquitous and important regulatory subunit) results in constitutive PKA activation (Bossis and Stratakis, 2004; Kirschner et al., 2005). Breeding of floxed *Prkar1a*, *Pkhd1*-Cre, and *Pkd1* ^RC/RC^ mice was used to generate mice with kidney specific constitutively active PKA on a *Pkd1* hypomorphic background and on a wild-type background (Ye et al., 2017). Constitutive kidney specific PKA activation stimulated multiple downstream signaling pathways (Src, Ras, ERK1/2, mTOR, GSK3β, β-catenin) and transcription factors (CREB, Stat3, Pax2) and markedly aggravated PKD. By itself constitutive kidney specific PKA activation on a wild-type background stimulated the same downstream signaling pathways and transcription factors and was sufficient to induce cystogenesis and fibrosis without much kidney enlargement. The presence of kidney and liver cysts in patients with the Carney complex, an autosomal dominant multitumoral disease caused by a heterozygous mutation in *PKAR1A*, supports the relevance of these observations for human ADPKD (Ye et al., 2017). More recently, mice with a conditionally expressed *Prkar1a* mutation (that makes RIα unable to release catalytic subunits in the presence of cAMP), *Pkhd1*-Cre and *Pkd1* ^RC/RC^ mice were used to generate mice with a constitutive kidney specific downregulation of PKA and their controls (Wang et al., 2022). Constitutive kidney specific PKA downregulation inhibited the same downstream signaling pathways and transcription factors activated by PKA constitutive activation and attenuated PKD.

Although these results provide a strong rationale for the utilization of PKA inhibitors to treat PKD, this has not been feasible because available PKA inhibitors are poorly selective and unsuitable for *in vivo* studies (Murray, 2008). Recently, highly specific and potent PKA inhibitors have been developed by Blueprint (Schalm et al., 2022). One of these compounds ameliorated the development of PKD in the *Pkd1* ^RC/RC^ with less increase in urine output compared to tolvaptan (Wang et al., 2022).

## Signal transduction kinases regulated by PKA

**FIGURE 4:**
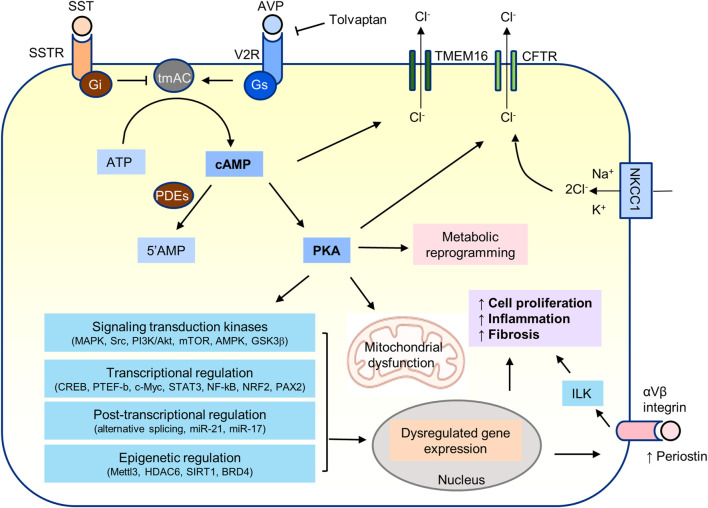
cAMP/PKA signaling in PKD.

Many signal transduction kinases have been shown to play a role in the pathogenesis of PKD. Cyclic AMP/PKA signaling may directly or indirectly regulate most of them (Figure 4).

### MAPK pathway

The classical MAPK signalling pathway is implicated in growth-factor and tyrosine kinase receptor-mediated cell proliferation. It consists of a cascade of three consecutive phosphorylation steps exerted by MAP3Ks (RAF proteins), MAP2Ks (MEK1/2), and MAPKs (ERK1/2) (Cuarental et al., 2019). Activated ERK1/2 translocate to the nucleus, where they phosphorylate and stabilize several transcription factors that are involved in the early phases of the G1–S cell cycle transition and the upregulation of glucose transporters and several rate-limiting glycolytic enzymes. In wild-type renal epithelial cells, PKA phosphorylates and inhibits Raf1 and MAPK signaling. In ADPKD cells or with calcium deprivation (lowering extracellular calcium concentrations or using calcium channel blockers) PKA increases the expression and activity of B-Raf in a Ras and Src dependent manner bypassing the inhibition of Raf-1 (Yamaguchi et al., 2004). It was proposed that calcium deprivation causes this phenotypic switch by inhibiting of PI3K/Akt signaling which in turn increases the levels of B-Raf levels via adjustments in synthesis and/or turnover rate.

Targeting Raf/MEK/ERK has given inconsistent results, possibly due to redundancies with other pathways. PLX5568 (Buchholz et al., 2011a), a Raf kinase inhibitor, attenuated cyst enlargement *in vitro* and in cy/+ Han:SPRD rats, but had no effect on kidney/body weight ratio or kidney function and promoted hepatic and renal fibrosis. Sorafenib, a Raf kinase inhibitor with activity against vascular endothelial growth factor receptor and platelet-derived growth factor receptor kinases, inhibited cAMP-dependent activation of B-Raf/MEK/ERK signaling, cell proliferation, and growth of ADPKD cysts *in vitro*. In contrast, sorafenib stimulated pERK1/2 and proliferation of *Pkd2* knockout cells *in vitro* and augmented ERK activation, cell proliferation and hepatic cystogenesis in *Pkd2* knockout mice (Yamaguchi et al., 2010; Spirli et al., 2012). Assays of Raf kinase activity showed that sorafenib inhibited B-Raf in wild-type and *Pkd2* knockout cells and inhibited Raf-1 in wild-type cells, but stimulated Raf-1 in *Pkd2* knockout cells. Pre-treatment with a PKA inhibitor or co-treatment with octreotide abolished the paradoxical activation and inhibited cyst growth. The MEK inhibitor PD184352 ameliorated PKD in pcy mice (Omori et al., 2006), but the MEK inhibitor UO126 had no effect on cystogenesis in *Pkd1* knockout mice at doses sufficient to reduce phospho-ERK1/2 in cystic kidneys (Shibazaki et al., 2008).

### Non-receptor tyrosine kinase Src

PKA phosphorylates c-Src on serine-17 to regulate its activity. Being a common link downstream from cAMP/PKA and the tyrosine receptor kinases pathway, Src is an attractive therapeutic target for ADPKD. Bosutinib, a Src/Abl inhibitor, was found to ameliorate PKD in bpk and *Pkd1* heterozygous mice and in PCK rats (Sweeney et al., 2008; Elliott et al., 2011). A phase II clinical trial (NCT01233869) showed a reduction in the rate of kidney growth but has not been further pursued for the treatment of ADPKD because of lack of effect on kidney function and high rate of discontinuation due to frequent adverse events (Tesar et al., 2017). Tesevatinib, an inhibitor of Src and multiple tyrosine kinase receptors (EGFR, ERBB2, and VEGFR2), ameliorated PKD and liver cystic disease in bpk mice and PCK rats. Phase I clinical trials have been completed and a phase II trial is ongoing, but no results have been published.

### Phosphoinositide 3-kinase and AKT serine/threonine kinase

PI3Ks are activated by receptor tyrosine kinases and phosphorylate membrane-associated phosphatidylinositol-4,5-bisphosphate to yield phosphatidylinositol-3,4,5-triphosphate (PIP3) (Margaria et al., 2020). PIP3 activates 3-phosphoinositide-dependent protein kinase 1 (PDK1), which in turn phosphorylates and activates Akt. The activity of PI3K is opposed by PTEN (Phosphatase and Tensin Homolog), which dephosphorylates PIP3 back to PIP2. PI3K and Akt stimulate cell proliferation via tuberin phosphorylation and activation of mTORC1. PI3K and Akt families consist of multiple members, eight (divided in three classes) and three respectively, with distinct functions. While cystic tissues from patients and rodent models of PKD exhibit enhanced phosphorylation of PI3K and Akt and PI3K/AKT signaling may promote cystogenesis (Wahl et al., 2007; Conduit et al., 2020), inhibition of PI3K/Akt signaling has also been proposed to be responsible for the proliferative response of the cystic epithelium to cAMP (Yamaguchi et al., 2004). Furthermore, class II PIK3C2A has been shown to inhibit cystogenesis by promoting correct cilia formation and targeting of polycystin-2 (Franco et al., 2016). Both genetic and pharmacologic downregulation of PKA have been shown to inhibit Akt phosphorylation in *Pkd1* ^RC/RC^ mice, possibly through interference in one or more pathways associated with AKT activation or inhibition of PTEN downstream from PKA (Wang et al., 2022).

### Mammalian target of rapamycin

mTOR functions as two distinct PKD multi-protein kinase-signaling complexes, mTORC1 and mTORC2 (Margaria et al., 2020). Downstream from PKA, ERK mediated phosphorylation and Akt activate mTORC1 in PKD (Rowe et al., 2013). Activated mTORC1 phosphorylates and activates 4E-BP (eukaryotic translation initiation factor 4E-binding protein) and S6K (S6 kinase) and controls mRNA translation, mitochondrial activity and biogenesis, and metabolic reprogramming (Hsieh et al., 2012; Thoreen et al., 2012; Morita et al., 2013). ERK-dependent inhibition of LKB1 (liver kinase B1) inhibits AMPK, which may further enhance mTORC1 signaling. At doses and blood levels achievable in humans, mTORC1 inhibiting rapalogs (sirolimus and everolimus) ameliorated PKD in cy/+ Han:SPRD rats, a model affecting proximal tubules, but not in PCK rats, a model affecting the distal nephron and collecting duct (Shillingford et al., 2006). Mice tolerate much higher doses and blood levels than rats and humans. These high doses of rapalogs were consistently effective in orthologous and non-orthologous mouse models. However, the results of clinical trials have been mostly discouraging (NCT00346918; NCT00491517; NCT00414440) (Serra et al., 2010; Walz et al., 2010), likely because achievable blood levels capable of inhibiting mTOR in peripheral blood mononuclear cells do not inhibit mTOR in the kidney or because mTORC1 inhibition triggers a compensatory activation of mTORC2, PI3K and Akt (Canaud et al., 2010).

### AMP-activated protein kinase

AMPK is a sensor of the cellular energy status reflected by the ratio AMP and ADP to ATP. AMP and ADP activate AMPK through an allosteric effect that facilitates its phosphorylation LKB1 and maintain AMPK in an active state by blocking its dephosphorylation by phosphatases. AMPK stimulates oxidative phosphorylation and fatty acid oxidation and inhibits aerobic glycolysis via activation of the tuberin/hamartin complex and inhibition of mTORC1. Drugs and xenobiotics that activate AMPK may be beneficial in PKD by inhibiting cell proliferation and chloride driven fluid secretion (Hallows et al., 2003). Metformin activates AMPK indirectly by inhibiting mitochondrial respiration (El-Mir et al., 2000). The results of preclinical studies of metformin for PKD have given inconsistent results. Metformin has been reported to be protective on *in vitro* and *ex vivo* renal cystogenesis, and *in vivo* in two rapidly progressive and in one slowly progressive *Pkd1* mouse models (Takiar et al., 2011; Pastor-Soler et al., 2022). In contrast metformin was detrimental in hypomorphic *Pkd1* miRNA transgenic mice (Chang et al., 2022). Another study compared two indirect (metformin and canagliflozin) and one direct (salsalate) AMPK activators in an inducible adult onset *Pkd1* knock-out mouse model (Leonhard et al., 2019); only salsalate had a protective effect. In the PCK, rat metformin ameliorated the liver disease but had no effect on the kidney disease (Sato et al., 2021). Oral metformin treatment was reported to slow PKD progression in a miniature pig model (Lian et al., 2019). Surprisingly, mouse models with a global or kidney specific expression of constitutively active AMPK, with a gain-of-function mutation in the AMPKγ1 subunit, resulted in an early-onset polycystic kidney phenotype with collecting duct cysts, compromised renal function, increased cAMP levels, ERK activation and hexokinase I expression (Wilson et al., 2021). Another study described cystic kidneys and impaired kidney function in mice expressing an activating mutation in the γ2 subunit of AMPK (Yang et al., 2016). Possibly, the timing (initiating versus progressing phases of the disease) and level of AMPK activation may account for these conflicting results.

The results of two phase II double-blinded randomized placebo-controlled trials, the TAME PKD (Trial of Administration of Metformin in PKD, NCT02656017) at Tufts University and University of Maryland in 97 adult patients with eGFR >50 ml/min/1.73 m^2^ treated for 2 years (Perrone et al., 2021) and the second (Feasibility Study of Metformin Therapy in ADPKD, NCT02903511) in the University of Colorado in 51 adult patients with eGFR 50–80 ml/min/1.73 m^2^ treated for 1 year (Brosnahan et al., 2022) have been recently reported. Both studies found that the administration of metformin was safe and tolerable. Changes in total kidney volume and eGFR in these underpowered studies were not significantly different between the groups.

### Glycogen synthase kinase β

GSK3β is a ubiquitously expressed and constitutively active serine/threonine protein kinase (Beurel et al., 2015). In normal mouse kidneys, GSK3β positively regulate cAMP generation in response to AVP (Rao et al., 2010). Mice with renal collecting duct-specific gene knockout of GSK3β have reduced adenylate cyclase activity, cAMP generation, and ability to concentrate urine. A positive feed-forward mechanism has been described whereby cAMP and CREB-mediated signaling stimulate GSK3β expression and GSK3β in turn enhances cAMP generation and CREB activity (Kakade et al., 2016). In mouse models of PKD, GSK3β expression increases progressively with age in parallel with the increase in renal cAMP levels. Collecting duct-specific gene knockout of GSK3β or treatment with the specific GSK3β inhibitor TDZD-8 (4-Benzyl-2-methyl-1,2,4-thiadiazolidine-3,5-dione) ameliorated PKD in cpk and kidney specific *Pkd1* knockout mice (Tao et al., 2015). This was accompanied by a significant reduction cyclin-D1 and c-Myc, whereas β-catenin levels were increased. The increase in β-catenin levels, which may be due to the inhibition of GSK3b that negatively regulates its cytoplasmic accumulation, suggests that β-catenin may not be critical for proliferation of the cyst-lining epithelium in these animal models.

## Regulation of ion channels relevant to polycystic kidney disease

### Cystic fibrosis transmembrane conductance regulator

Active transport of chloride from the basolateral to the apical side is the driving force for fluid secretion into the cysts (Figure 4) (Sullivan et al., 1998; Grantham, 2003; Jouret and Devuyst, 2020). The energy is generated by the sodium pump (Na, K-ATPase) in the basolateral membrane of cyst epithelial cells. Chloride enters from the basolateral side through the sodium-potassium-chloride cotransporter (NKCC1) and uses the gradient established by the sodium pump to bring potassium and chloride into the cells. PKA-induced phosphorylation of the cystic fibrosis transmembrane conductance regulator (CFTR) in the apical membrane opens the channel and allows the flow of chloride ions down an electrochemical gradient into the cyst, generating increased transepithelial electron activity that, in turn, drives sodium ions through paracellular pathways. Cyclic AMP and PKA signaling also promote the surface expression of NKCC1 in the thick ascending limb of Henle and of functional Na,K-ATPase units in cortical collecting duct principal cells (Kim et al., 1999; Gonin et al., 2001; Ortiz, 2006).

CFTR blockers (glibenclamide, NPPB, genistein) have been shown to reduce cyst growth and cAMP stimulated chloride currents in MDCK cyst in collagen gel (Magenheimer et al., 2006; Lu et al., 2010). CFTR thiazolidinone inhibitors stabilize the channel in the closed state and inhibited cystogenesis *in vitro*, *ex vivo* (metanephric kidney organ culture), and *in vivo* (rapidly progressive kidney-specific *Pkd1* knock-out mice (Yang B. et al., 2008; Snyder et al., 2011)). Steviol has been reported to promote CFTR degradation by the proteasome, inhibit MDCK cystogenesis *in vitro* and in kidney specific *Pkd1* knockout mice treated with high intraperitoneal doses (Yuajit et al., 2013; Yuajit et al., 2014; Nantavishit et al., 2018). The proton pump inhibitor Lansoprazole has also been recently proposed to reduce kidney cysts *in vitro* (MDCK cells) and *in vivo* (PCK rat) via activation of the liver X receptor and subsequent down-regulation of CFTR (Nantavishit et al., 2018). Treatment of *Pkd1* knockout mice with the CFTR regulator VX-809 (Lumacaftor) increased the localizations of CFTR in the basolateral membrane of cyst lining cells and those of the sodium proton exchanger 3 and the epithelial sodium channel in the apical membrane, thus promoting net resorption of cyst fluid, reducing cyst growth, and protecting kidney function (Yanda et al., 2018). A phase 2, placebo-controlled randomized controlled trial (NCT04578548) to investigate the safety and tolerability of the CFTR inhibitor GLPG2737 in ADPKD patients at risk for rapidly progressive disease((U.S.), 2020) is ongoing.

### TMEM16A (anoctamin-1)

Recent studies suggest that the calcium-dependent chloride channel TMEM16A may also promote chloride driven fluid secretion into the cysts (Buchholz et al., 2011b; Buchholz et al., 2014; Kraus et al., 2016; Schreiber et al., 2019). It is upregulated in the apical membrane of human cyst-lining cells and may be activated by ATP in the cyst fluid acting on P2Y receptors (Buchholz et al., 2011b). Apyrase (ATP scavenger) and suramin (P2 receptor inhibitor) reduced cAMP-driven fluid secretion in MDCK cysts while increasing extracellular ATP potentiated cAMP-mediated cyst growth, suggesting a synergistic interaction between CFTR and TMEM16A. TMEM16A inhibitors and morpholinos inhibited cyst growth in metanephric kidney cultures (Buchholz et al., 2014). Tubule-specific knockout of TMEM16A and TMEM16A inhibitors ameliorated PKD in an adult *Pkd1* orthologous mouse model (Cabrita et al., 2020). In contrast, the effect of deleting of CFTR together with *Pkd1* was not statistically significant in a small number of adult mice (5 and 7) (Talbi et al., 2021). The presence of TMEM16A was reported to be necessary for the expression of CFTR at the plasma membrane. It has been suggested that TMEM16A activation, linked to local hypoxia and stabilization of the hypoxia-inducible transcription factor-1α (HIF-1α) promoting the expression of P2YR, may be more important at advanced than at early stages of PKD (Kraus et al., 2018).

## PKA regulation of extracellular matrix

Alterations in focal adhesion complexes, basement membranes, and extracellular matrix contribute to the pathogenesis of PKD. Focal adhesion complexes contain integrin αβ heterodimer receptors, which link the actin cytoskeleton to basement membrane laminin αβγ heterotrimers, collagens and matrix proteins. Abnormal expression of these proteins accelerates cyst growth through activation of integrin signaling (Daikha-Dahmane et al., 1997; Joly et al., 2003; Shannon et al., 2006; Wu et al., 2009).

Periostin is a secreted matricellular protein that binds to αVβ3 and αVβ 5 integrins and is expressed during development and tissue remodeling. In a microarray analysis of cultured human ADPKD cyst epithelial cells, periostin mRNA was markedly overexpressed compared with normal human kidney cells (Wallace et al., 2008). Periostin overexpression, which is important for the development of myocardial infarction induced myocardial fibrosis, can be prevented by siRNA or shRNA CREB downregulation both *in vitro* and *in vivo* (Xue et al., 2022). It seems likely that the overexpression of periostin in cystic kidneys is also driven by CREB likely downstream from PKA signaling (Figure 4).

ADPKD cells secret periostin across luminal and basolateral plasma membranes. Periostin binds to αVβ3 and αVβ integrins, activates the integrin-linked kinase (ILK), a component of focal adhesion plaques, and promotes *in vitro* cyst growth (Wallace et al., 2008). Pharmacologic inhibition or shRNA knockdown of ILK prevented periostin-induced Akt/mTORC1 signaling and ADPKD cell proliferation *in vitro*. Knocking out periostin (Postn) inhibited mTOR signaling, cell proliferation and interstitial fibrosis, and ameliorated PKD in pcy mice (Wallace et al., 2014). Lowering the expression of ILK had similar effects in rapid and slowly progressive kidney-specific *Pkd1* knockouts. Whereas heterozygous knockdown of ILK in collecting ducts of wild-type mice had no effect on renal morphology or function, complete ILK knockout caused caspase-3 mediated anoikis, dilated cortical tubules with apoptotic cells, interstitial fibrosis, and death by 10 weeks of age (Raman et al., 2017). Because complete knockout of ILK in collecting duct cells caused renal injury, long-term use of an ILK inhibitor may not be feasible.

## Transcriptional regulation downstream from PKA

Many transcription factors implicated in the pathogenesis of PKD are regulated by cAMP and PKA signaling among other pathways (Figure 4).

### Cyclic AMP response element-binding protein

Under basal conditions the regulatory and catalytic PKA subunits colocalize in cytoplasmic puncta (Zhang et al., 2020). In stimulated cells, cAMP generated by internalized agonist-bound Gαs protein-adenylyl cyclase complexes in endosomes diffuses to adjacent puncta releasing the catalytic subunits that enter nuclei and phosphorylate substrates involved in transcriptional regulation. PKA phosphorylates and activates the cAMP responsive element binding protein (CREB) family of transcription factors, CREB1, cAMP responsive element modulator (CREM), and activating transcription factor 1 (ATF-1) (Mayr and Montminy, 2001; Rosenberg et al., 2002). Phosphorylated CREB recruits the coactivators CREB binding protein (CBP) or p300 and bind to cAMP-response elements (CREs) in the genome to drive transcription of target genes. CBP and p300 are histone acetyltransferases that enhance the ability of CREB to activate transcription by relaxing the chromatin structure at gene promoter regions and creating scaffolds for recruitment of RNA polymerase II complexes to the promoter.

CREB is phosphorylated and hyperactive in ADPKD (Ye et al., 2017). An integrative analysis using cleavage under targets and release using nuclease (CUT&RUN), RNA-sequencing, and rescue of differentially expressed genes by treatment with a selective CREB inhibitor (665-15) identified the genomic loci bound to phosphorylated CREB in cystic epithelial cells (Liu et al., 2021). Function enrichment analysis of CREB direct targets revealed prominent enrichment of genes related to cell proliferation and inflammation-related pathways, including ribosome biogenesis, metabolism of RNA, the tight junction, metabolism of polyamines, the cell cycle, and the immune response. Treatment with 666-15 and overexpression of a dominant-negative inhibitor of CREB (A-CREB) were protective in mice with an inducible *Pkd1* knockout (Liu et al., 2021). Novel CREB inhibitors with higher solubility and bioavailability have been actively under development and shown promise in preclinical studies (Sapio et al., 2020).

### Positive transcription elongation factor b and CREB regulated transcription coactivator 2

PTEFb and CRTC2 are also critical to regulate transcription mediated by CREB and other transcription factors. Both are activated by PKA. Under basal conditions PTEFb is kept inactive in a silencing P-TEFb/HEXIM1/7SK snRNP complex (Yik et al., 2003; Michels et al., 2004). P-TEFb is hyperactivated in mouse and human ADPKD kidneys (Sun Y. et al., 2019). PKA phosphorylates HEXIM1 and releases PTEFb which is then recruited to target genes by direct interaction with transcription factors. These include transcription factors of great importance in the pathogenesis of PKD such as c-Myc, Stat3, and NFkB. Constitutive activation of PTEFb induces pronephric cysts in zebrafish and the pTEFb inhibitor flavopiridol ameliorates the cystic disease in rapid and slowly progressive *Pkd1* mouse models. PKA also activates P-TEFb through the activation of CRTC2 (Mi et al., 2022). Under basal conditions CRTC2 is phosphorylated by salt inducible kinase 1 (SIK1) and retained in the cytoplasm through phosphorylation-dependent interactions with 14-3-3 proteins. PKA phosphorylation inactivates SIK1 and allows the translocation of CRTC2 to the nucleus where it forms liquid-liquid condensates and activates P-TEFb by disrupting the inhibitory 7SK snRNP complex. Genetic depletion of CRTC2 suppresses cyst growth in an orthologous ADPKD mouse model.

### c-Myc proto-oncogene

c-Myc is overexpressed in the kidneys of human as well as of virtually all orthologous and non-orthologous animal models of PKD (Kurbegovic and Trudel, 2020). The induction of PKD by renal overexpression of c-Myc in transgenic mice and the amelioration of the disease by its genetic or pharmacologic downregulation demonstrate a causal connection between c-Myc and cystogenesis. The precise molecular mechanism(s) responsible for c-Myc activation in ADPKD is not known but may involve cAMP/PKA signaling. C-Myc is regulated by multiple mechanisms at the transcriptional, translational, and post-translational levels. PGE2 acting on an EP4R/GS/AC/cAMP/PKA/CREB signaling pathway increases c-Myc expression at both mRNA and protein levels and proliferation of hepatocellular carcinoma cells (Xia et al., 2014). PKA has also been shown to protect c-MYC from proteasome-mediated degradation through phosphorylation at Ser-279 in prostatic carcinoma cells (Padmanabhan et al., 2013).

### Signal transducer and activator of transcription 3

STAT3 is a member of the STAT family of transcriptions factors. STAT3 activation mediates promotes migration of neutrophils, B-lymphocytes, dendritic cells and macrophages, and inflammation (Strubl et al., 2020). It is strongly activated in renal cyst-lining cells in human ADPKD and several PKD mouse models (Strubl et al., 2020). In addition to members of the Janus family of protein kinases (JAK) and several tyrosine kinase receptors (EGFR, PDGFR and c-Met), the non-receptor tyrosine kinase Src phosphorylates and activates STAT proteins promoting their nuclear translocation (Wang et al., 2000; Silva, 2004). Cyclic AMP/PKA signaling was found to enhance the effect of the cleaved PC1 tail Src and STAT3 activation (Talbot et al., 2014). In the PCK rat model, activation of STAT3 in renal cystic cells depended on vasopressin V2R signaling. Genetic inhibition of vasopressin expression or treatment with a pharmacologic V2R inhibitor strongly suppressed STAT3 activation and reduced renal cyst growth (Wang et al., 2008). Two STAT3 inhibitors, pyrimethamine and S3I-201, also inhibited cyst growth in a neonatal and an adult *Pkd1* model (Takakura et al., 2011). Curcumin, a compound with a broad spectrum of activity that ameliorates PKD, also inhibits STAT3 (Leonhard et al., 2011).

### Nuclear factor κB

The NF-κB family of transcription factors consists of NF-κB1 (also named p50), NF-κB2 (also named p52), RelA (also named p65), RelB and c-Rel, which form different combinations of homo- or heterodimers (Barnabei et al., 2021). These dimers (e.g., p65:p50) are retained in the cytoplasm while bound to IκB proteins. Pro-inflammatory stimuli (e.g., TNFα, lipopolysacharide, etc) activate IκB kinases (IKKs) that promote IκB phosphorylation, ubiquitination and proteolysis. Released NF-κB dimers can then translocate into the nucleus, bind to promoter sequences of inflammation-related genes, and recruit chromatin modifying coactivator complexes or components of the general transcriptional machinery (Bhatt and Ghosh, 2014). Phosphorylation of NF-κB subunits by multiple kinases may either enhance or downregulate the transcription of target genes (Christian et al., 2016). PKA dependent phosphorylation may either inhibit or promote NF-κB activity (Gerlo et al., 2011). PKA has been demonstrated to phosphorylate p50 at S337, which is critical for DNA binding, *in vitro* and *in vivo*. PKA also phosphorylates p65 at S276. In resting cells the PKA catalytic subunit is bound in an inactive state to cytosolic IκBα:p65 complexes. Following IKK complex activation and degradation of IκBα, the active PKA catalytic subunit is liberated and phosphorylates p65 at S276 in a cAMP-independent process. S276 phosphorylation triggers a conformational change in p65 that promotes its interaction with CBP/p300 and increases p65 transcriptional activity (Christian et al., 2016). Several studies support a role for NF-κB in cyst formation. *Pkd1* ^−/−^ cells and kidneys exhibit increased phosphorylation and nuclear localization of the NF-κB subunit p65 and NF-κB driven overexpression of Pax2 (paired box 2), Wnt7a and Wnt7b (Qin et al., 2012). Inhibition of NF-κB or Wnt7b ameliorates the cystic disease in organ culture models and *Pkd1* mutant mice. The increased expression of Wnt7a, Wnt7b and Pax2 downstream from NF-κB after the addition of 8-Br-cAMP does not prove but is consistent with cAMP-PKA signaling enhancing the activity of NF-κB in PKD.

### Nuclear factor erythroid 2–related factor 2

Nrf2 is an important regulator of antioxidant and anti-inflammatory mechanisms (Guerrero-Hue et al., 2020; Ito et al., 2020). Under physiological conditions the Kelch-like ECH-associated protein 1 (Keap) binds to Nrf2, facilitates its ubiquitination and proteasomal degradation, and prevents its translocation to the nucleus (Zhang D. D. et al., 2004). Reactive oxygen species disrupt the interaction and facilitate the nuclear translocation, transcriptional upregulation of antioxidant enzymes and transcriptional downregulation of inflammatory cytokines. A second mechanism to prevent Nrf2 signaling is phosphorylation by GSK3β that also facilitates ubiquitination and proteasomal degradation (Lu et al., 2019). In PKD, at least in advanced stages, the expression of KEAP and GSK3β are increased and Nrf2 expression is low (Lu et al., 2020). Deletion of Nrf2 further aggravates the severity of the PKD. In contrast, activation of Nrf2, either by Sulforaphane that disrupts the Nrf2-KEAP interaction, or by a compound that inhibits GSK3β, ameliorates PKD (Lu et al., 2020). A subanalysis of ADPKD patients in a clinical trial of the Nrf2 activator bardoxolone for CKD (Phoenix) showed that bardoxolone increased eGFR during a three-month follow-up (Pergola et al., 2019). A phase III clinical trial of bardoxolone in ADPKD is ongoing.

### Paired box 2

Pax 2 upregulation contributes to the proliferative phenotype of the cystic epithelium. Pax2 is highly expressed in developing collecting ducts and the cystic epithelium (Dressler and Woolf, 1999). Heterozygous Pax2 mutants, mice and humans, have a phenotype due to a loss of gene dosage consistent with haploinsufficiency. A reduction of Pax2 gene dosage slowed the progression of PKD in two different mouse models of PKD (*Pkd1* mutant and cpk) (Ostom et al., 2000; Stayner et al., 2006). Conversely, transgenic overexpression of Pax2 results in cyst formation in kidneys (Dressler et al., 1993; Stayner et al., 2006). Addition of 8-Br-cAMP increased the expression of Pax2 in *Pkd1* ^−/−^ explants but not in Pkd1 WT kidneys. Constitutive activation or inhibition of PKA in Pkd1^RC/RC^ mice increase or decrease Pax2 expression respectively. These observations suggest the expression of Pax2 in PKD is at least in part downstream from PKA. Pax2 has recently been shown to be part of a H3K4 methyltransferase complex, which is known to activate gene expression (Patel et al., 2007).

## Post-transcriptional regulation downstream from PKA

### Alternative splicing

Alternative splicing is controlled by intronic and exonic regulatory elements called intronic or exonic splicing enhancers or silencers. PKA phosphorylates arginine/serine-rich splicing factors and has been implicated in the regulation of alternative splicing (Figure 4) (Li et al., 2009). The control of alternative splicing by PKA and CaMKIV acting on the same RNA elements may be another point of convergence between cAMP and calcium signaling in PKD. The role of alternative splicing has been studied in cancer and many other diseases but not in PKD (Climente-Gonzalez et al., 2017; Goncalves et al., 2017). In cancer the proliferative or anti-proliferative responses to cAMP have been reported to depend on the relative expression of two B-Raf splicing variants (95 and 62 kD) (Papin et al., 1998; Fujita et al., 2002). cAMP stimulates cell proliferation in cells that express mostly the 95-kD isoform, whereas it is inhibitory in cells that do not express B-Raf or express mostly the 62-kD isoform. The relative expression of these two isoforms depends not only on cell type but also on cellular density (predominantly 95-kD isoform in subconfluent cells and 62-kD isoform in confluent cells) and may work as a molecular switch to activate and inhibit ERK and cell proliferation (Takahashi et al., 2004). Interestingly, the protective effect of two V2R antagonists in PKD models was accompanied by a significant reduction in the ratio of the 95- and 62-kD isoforms (Wang et al., 2005).

### Non-coding RNAs

The importance of non-coding RNA for the regulation of the expression of mRNAs is now well recognized. MicroRNAs (miRNAs) are ∼22 nucleotides double-stranded non-coding RNAs (ncRNAs) that function as inhibitors of post-transcriptional mRNA expression. Numerous miRNAs are aberrantly expressed in murine and human forms of ADPKD (Ramalingam et al., 2020).

miR-21 is amongst the most upregulated miRNA in response to tissue injury (Ramalingam et al., 2020). It is thought to mediate repair and regeneration, but sustained miR-21 activation is maladaptive and can propagate injury and tissue fibrosis. cAMP-PKA signaling transactivates the miR-21 gene via conserved CREB motifs in the miR-21 promoter region (Figure 4) (Lakhia et al., 2016). miR-21 is overexpressed in PKD (Lakhia et al., 2016; Woo et al., 2017). miR-21 deletion attenuates PKD progression in the *Pkd2*-KO mouse model. The V2R antagonist mozavaptan inhibits the cAMP-PKA pathway and reduces miR-21 expression in ADPKD mouse models. The validated targets of miR-21 are well-known players of survival and inhibitors of apoptosis (Buscaglia and Li, 2011). Pdcd4 (programmed cell death 4) and Ppara are among the many genes downregulated by miR-21 (Lakhia et al., 2016). Pdcd4 mediates apoptosis and Ppara promotes fatty acid oxidation. Repression of apoptosis and fatty acid oxidation are thought to play a role in the pathogenesis of PKD.

miR-17∼92 is a polycistronic miRNA cluster, which consists of six individual miRNAs (miR-17, miR-18a, miR-19a, miR-20a, miR-19b-l, and miR-92a) (Ramalingam et al., 2020). It is essential for the development of the kidneys and many other organs, but its expression declines with maturation, its inducible deletion in adult mice and kidney specific deletion after nephrogenesis have no impact (Ramalingam et al., 2020). c-Myc transactivates miR-17∼92 via conserved binding sites in the miR-17 promoter region. Transgenic upregulation of c-Myc or miR-17∼92 in wild-type mice causes PKD (Patel et al., 2013). Genetic deletion of miR-17∼92 attenuates PKD in multiple mouse models (Patel et al., 2013). Anti-miRs against the individual miRNA families of the six-member miR-17∼92 cluster were developed to identify the family that is the crucial pathogenic driver in PKD. Anti-miR-17, but not antimiR-18, anti-miR-19 or anti-miR-25 LNAs, attenuated cyst growth in *Pkd1* knock-out mice (Yheskel et al., 2019). Anti-miR-17 derepressed many mRNA targets including Ppara, *PKD1*, and *PKD2*. These observations have provided the scientific rationale for targeting the miR-17 family for ADPKD treatment.

RGLS4326, a single-stranded, phosphorothioate oligonucleotide of only 9 nucleotides in length that bears complementarity to the miR-17 seed sequence, was synthesized for investigation in clinical trials (Lee et al., 2019). RGLS4326 displaces miR-17 from translationally active polysomes and thereby de-represses multiple miR-17 mRNA targets. RGLS4326 treatment attenuates cyst growth in multiple PKD mouse models and human *in vitro* ADPKD models. A Phase 1b, open-label, adaptive design dose-ranging study to evaluate ADPKD biomarkers, pharmacokinetics, safety, tolerability, and pharmacodynamics of RGLS4326 administered via SC injection to patients with ADPKD was terminated early to prioritize a different compound.

## Epigenetic mechanisms downstream from PKA

Epigenetic regulation is the process that modifies gene expression in the absence of changes in genome sequence. The main mechanisms of epigenetic regulation are DNA and RNA methylation, and histone modification. Three main histone modifications are phosphorylation, methylation, and acetylation. Recent evidence suggests that epigenetic regulators play important roles in cyst growth in ADPKD. Epigenetic regulators have been reported to be downstream targets of cAMP/PKA/CREB signaling, and/or modulate this pathway (Figure 4).

### DNA methylation

DNA methylation is a major epigenetic mechanism that a methyl group was transferred onto the C5 position of the cytosine of CpG sites to form 5-methylcytosine (5 mC). This process is mediated by DNA methyltransferases (DNMTs), including DNMT3a, DNMT3b, and DNMT1 in mammals. DNMT3a and DNMT3b are associated with *de novo* DNA methylation during embryogenesis, whereas DNMT1 is involved in maintenance of methylation pattern (Moore et al., 2013). DNA methylation within a gene promoter typically suppresses gene expression through inhibiting the binding of transcription factors or recruiting histone deacetylases that results in chromatin condensation and gene inactivation (Moore et al., 2013). Methylation of CpGs within the gene body leads to increased expression of a gene (Jones, 2012).

The genome-wide DNA methylation status in kidneys from ADPKD and non-ADPKD individuals has been analyzed by performing methylated-CpG island recovery assay with parallel sequencing (MIRA-seq). The hypermethylation within the gene-body regions of *PKD1* and other genes associated with iron transport and cell adhesion were identified in ADPKD (Woo et al., 2014). The hypermethylation of *PKD1* led to its downregulation. Additionally, the methylation of *PKD1* promoter inversely correlates with its gene expression in peripheral blood of ADPKD patients (Hajirezaei et al., 2020). Inhibition of DNA methylation with 5-aza-2′-deoxycytidine increased the expression of *Pkd1* and decreased the cyst formation of MDCK cells (Woo et al., 2014). The CpG island within promoter region of MUPCDH gene was hypermethylated, which resulted in downregulation of MUPCDH and abnormal cell proliferation (Woo et al., 2015). The hypermethylation of MUPCDH promoter is correlated with the increased rate of total kidney volume change in ADPKD (Woo et al., 2015). In contrast, global hypomethylation was found in genomic DNA from ADPKD kidneys versus non-ADPKD kidneys by performing reduced representation bisulfite sequencing (RRBS) (Bowden et al., 2018). The hypermethylation was identified with 3′ end of *PKD1* gene body, but not associated with decreased expression of *PKD1* mRNA. A recent study investigated the DNA methylation changes of cystic epithelia within independent cysts by RRBS (Bowden et al., 2020). This study demonstrated that the greatest amount of variation occurs in fragments within CpG islands and gene bodies, but not within intergenic fragments across the ADPKD kidney. These regions with variation were defined as inter-cyst variants (ICVs). A proportion of the IVC associated genes were differentially methylated in ADPKD versus non-ADPKD kidney tissue. This work provided evidence that different DNA methylation changes contributes to the development of each cyst.

cAMP signaling has been reported to induce cardiac hypertrophy through regulating DNA methylation (Fang et al., 2015). Increased intracellular cAMP by the stable cAMP analog DBcAMP or PDE inhibitor caffeine and theophylline increased the expression of DNMTs and increased the global DNA methylation in HL-1 cardiomyocytes. Inhibition of DNMT activity with 5-azacytidine decreased global DNA methylation induced by DBcAMP.

DNA hydroxymethylation is a novel epigenetic modification of DNA. 5-methycytosine (5 mC) is converted to 5-hydroxymethylcytosine (5hmC) that is catalyzed by TET family proteins. TET proteins are Fe(II)-dependent and 2-oxoglutarate-dependent methylcytosine dioxygenases. 5hmC acts as an intermediate in the reaction of DNA demethylation or a signal for chromatin factors (Guibert and Weber, 2013). cAMP treatment increased the generation of 5hmC in multiple cell types (Camarena et al., 2017). cAMP promoted Fe(II) release to the intracellular labile Fe(II) pool (LIP) by the acidification of endosomes. This effect was confirmed by stimulation of GPCR adenylate, and by cyclase activators and PDE inhibitors. The DNA hydroxymethylation induced by cAMP was correlated with a majority of differentially transcribed gene. In addition to cAMP/PKA targeted transcription factors, cAMP modulates transcriptome by promoting DNA demethylation. DNA hydroxymethylation and demethylation have not been investigated in ADPKD. Whether cAMP contributes to cystogenesis via regulating DNA methylation and hydroxymethylation needs to be investigated in the future.

### RNA methylation

RNA methylation regulates the stability of mRNA and its translation to protein. The N6-methyladenosine (m6A) methylation of mRNA (m6A) is medicated by methyltransferase-like 3 (Mettl3). The levels of Mettl3 and m6A were upregulated in kidneys of mouse and human ADPKD (Ramalingam et al., 2021). Knockout of Mettl3 delayed cyst growth in three orthologous ADPKD mouse models. Overexpression of Mettl3 in a kidney specific transgenic mouse model developed tubular cysts. C-Myc and Avpr2 were identified as Mettl3 targets by methylated RNA immunoprecipitation sequencing (MeRIP-seq). Mettl3 promotes renal cystic epithelial cell proliferation by regulating the expression of c-Myc and Avpr2. m6A levels on c-Myc and Avpr2 mRNAs were upregulated in mouse PKD kidneys versus control kidneys, which were decreased in PKD1 and Mettl3 double knockout kidneys. The Mettl3 induced the phosphorylation of CREB via the upregulation of Avpr2 protein and activation of cAMP. In bone marrow mesenchymal stem cells, deletion of Mettl3 decreased cAMP accumulation induced by PTH and its downstream phosphorylation of CREB (Wu et al., 2018). One recent study reported that CREB upregulated the expression of miR-373 that suppressed Mettl3 in chondrocytes (Zhang et al., 2022). Whether cAMP/PKA/CREB signaling directly regulates the transcription of Mettl3 remains unclear.

### Histone deacetylases

The acetylation of histone tail is catalyzed by histone acetyltransferases (HATs). Histone acetylation mainly increases transcriptional activity via promoting the accessibility of transcriptional regulatory proteins to relaxed chromatin. Deacetylation of histone is medicated by histone deacetylases (HDACs). Deacetylation of histone leads to heterochromatin and inhibition of gene transcription. HDACs can also bind to and catalyze non-histone proteins. Human HDACs are divided into four categories: Class I HDAC (HDAC1, HDAC2, HDAC3, and HDAC8), Class II proteins (HDAC4, HDAC5, HDAC6, HDAC7, HDAC9, and HDAC10), Class III HDAC (SIRT1, SIRT2, SIRT3, SIRT4, SIRT5, SIRT6, and SIRT7), and Class IV HDAC (HDAC11) (Seto and Yoshida, 2014). Class I, II and IV HDACs are zinc-dependent deacetylase, while class III HDACs require NAD+ as the cofactor for deacetylase activity (Seto and Yoshida, 2014).

Trichostatin A (TSA), a class I and class II HDAC inhibitor, and valproic acid (VPA), a class I specific HDAC inhibitor, have been found to suppress kidney cyst formation by performing a chemical modifier screening in PKD zebrafish models (Cao et al., 2009). TSA ameliorates cyst formation through regulating different factors, such as Id2/p21 and Rb/E2F1 pathways(Fan et al., 2012), AMPK pathway, and autophagy (Sun L. et al., 2019). Additionally, HDAC5 has been identified as one of the targets of polycystin-dependent fluid stress sensing in renal epithelial cells (Xia et al., 2010). TSA modulates renal epithelial cell differentiation by targeting HDAC5 (Xia et al., 2010). Quisinostat, a second-generation class I and class II HDAC inhibitor, has been discovered to affect the viability of ADPKD cells with minimal effect on normal human kidney cells via a high-throughput screening platform of cancer drugs (Asawa et al., 2020).

The expression and activity of HDAC6 are upregulated in PKD mutant cells and kidneys (Liu et al., 2012b; Cebotaru et al., 2016). Targeting of HDAC6 with different specific inhibitors, including tubastatin, tubsin and ACY-1215 reduced cyst growth in PKD mouse models by inhibiting cystic renal epithelial cell proliferation (Cebotaru et al., 2016; Yanda et al., 2017a; Yanda et al., 2017b). Inhibition of HDAC6 reduced intracellular cAMP and intracellular calcium level by increasing ATP-stimulated calcium release and reducing the release of calcium from the endoplasmic reticulum (Yanda et al., 2017a; Yanda et al., 2017b). Treatment with tubacin decreased CFTR chloride currents activated by cAMP in MDCK cells (Cebotaru et al., 2016). Inhibition of HDAC6 by ACY-1215 reduced hepatic cystogenesis in polycystic liver disease model, PCK rats (Lorenzo Pisarello et al., 2018). A combination of ACY-1215 and the somatostatin receptor analogue, pasireotide synergistically reduced liver cyst growth in PCK rats. ACY-1215 and pasireotide decreased cAMP levels and cell proliferation in cultured cystic cholangiocytes. These findings support that the activation of HDAC6 promotes kidney and liver cyst growth and is an upstream regulator of cAMP in PKD. Isoproterenol activated cAMP/PKA via GPCR and inhibited downstream c-Raf/MEK/ERK signaling, and consequently increased the expression of HDAC6 in human lung cancer cells (Lim and Juhnn, 2016). Whether the activation of cAMP/PKA/CREB signaling in cystic renal epithelial cells regulates the expression and activity of HDAC6 to form a positive feedback loop has not been studied.

Several HDAC inhibitors have been approved by the FDA for the use in T cell lymphoma and multiple myeloma. The efficacy of the combination of HDAC inhibitor and different chemotherapy regimen in these hematological neoplasms were studied in several clinical trials (Bondarev et al., 2021). HDAC inhibitor alone or the combination of HDAC inhibitor and tolvaptan may be novel therapeutic strategies in ADPKD patients.

Sirtuin 1 (SIRT1) is the most conserved mammalian NAD+ dependent histone deacetylase. Activation of cAMP/PKA pathway by forskolin, an adenylyl cyclase activator, epinephrine, or a beta 2 adrenergic receptor agonist clenbuterol increased the deacetylase activity of SIRT1 via the phosphorylation of its serine 434 (Gerhart-Hines et al., 2011). The activation of SIRT1 increased PGC-1a deacetylation that results in the increased fatty acid oxidation (Gerhart-Hines et al., 2011). The transcription of SIRT1 is directly stimulated by the activation of cAMP/PKA/CREB as a response to humoral factors (glucagon and norepinephrine) released during fasting (Noriega et al., 2011). Seven putative half CREB-binding sites are identified in the proximal promoter region of SIRT1 gene. Stimulation of cAMP by forskolin, glucagon, and norepinephrine increased the SIRT1 mRNA abundance in hepatic cells (Noriega et al., 2011). A recent study in *C. elegans* reported that the activation of PKA by hydralazine was mediated by its binding and stabilization of a catalytic subunit of PKA, which was independent of cAMP (Dehghan et al., 2019). PKA activated the downstream SIRT1 and SIRT5, leading to improved mitochondrial function and metabolic homeostasis (Dehghan et al., 2019). Furthermore, CREB promotes SIRT1 transcription via binding to its promoter in neuronal cells (Fusco et al., 2012). CREB recruits SIRT-1 to DNA, which leads to the increased expression of CREB target genes, such as Peroxisome proliferator-activated receptor-γ coactivator 1-α (PGC1α) and neuronal NO synthase (Fusco et al., 2012).

The expression of SIRT1 was upregulated in *Pkd1* mutant mouse renal epithelial cells and kidney tissues, and human ADPKD cells (Zhou et al., 2013; Warner et al., 2016). SIRT1 expression is regulated by c-Myc and induced by TNFa. Conditional double knockout of SIRT1 and Pkd1 delayed cyst growth in *Pkd1* ^flox/flox^:Ksp-Cre mice (Zhou et al., 2013). Inhibition of SIRT1 with a pan-sirtuin inhibitor nicotinamide and a specific inhibitor EX-527 slowed cyst growth in three PKD mouse models (embryonic model *Pkd1* ^−/−^, *Pkd1* ^flox/flox^:Ksp-Cre, and hypomorphic *Pkd1* ^nl/nl^). The upregulated SIRT1 increased cystic renal epithelial cell proliferation through deacetylation of Rb, and inhibited apoptosis via deacetylation of p53. The SIRT1 activator resveratrol treatment increased cyst formation, and FK866, an NAD synthetic enzyme Nampt inhibitor, decreased cyst formation in the cystogenic assay of MDCK cells (Warner et al., 2016). The expression of SIRT2 was upregulated in *Pkd1* knockout mouse kidney cells. SIRT2 is a NAD+ dependent protein deacetylase that interacts with HDAC6 and deacetylates a-tubulin (North et al., 2003). SIRT2 regulates cilia formation and disassembly and contributes to aberrant centrosome amplification and polyploidy in PKD (Zhou et al., 2014).

These preclinical studies have provided the rationale to support that SIRT1 is an attractive therapeutic target of ADPKD. Nicotinamide (Niacinamide), also known as vitamin B3, is a water-soluble amide derivative of nicotinic acid and a dietary supplement. Nicotinamide inhibits sirtuins at high doses. In a small randomized, double blinded, placebo-controlled trial, nicotinamide is safe and well-tolerated in patients with ADPKD but does not show difference in the change in height-adjusted total kidney volume over 12 months (El Ters et al., 2020). Due to the limitations of small sample size and short duration of intervention, further studies will be needed to validate nicotinamide as a clinical therapy for ADPKD. EX-527 (Selisistat), a SIRT1 specific inhibitor, ameliorated PKD in three ADPKD mouse models. EX-527 has been found to be well-tolerated and safe in healthy human subjects (Curry et al., 2021). Two phase III clinical trials of EX-527 for treatment of Huntington’s disease and endometriosis-mediated IVF failure respectively are ongoing. EX-527 is a potential therapy for ADPKD patients that needs to be studied.

### Bromodomain-containing protein 4

BRD4, a member of bromodomain and extraterminal (BET) proteins, is an epigenetic reader that binds to acetylated lysine residue on histones. BRD4 activates gene transcription through the recruitment of p-TEFb and multiprotein mediator complex. BRD4 has been found to be involved in transcriptional activation by cAMP/PKA/CREB pathway. CREB typically activates the transcription of its target genes by recruitment of coactivators to promoter proximal binding sites. CREB regulates the expression of pancreatic beta cell-specific genes through hyperacetylation of promoter-distal super-enhancer region (Van de Velde et al., 2019). CREB recruits histone acetyltransferase p300 and CREB binding protein (CBP) to super-enhancer region, subsequently increases in histone acetylation and facilitates the recruitment of the coactivators CREB-regulated transcription coactivator 2 (CRTC2) and BRD4 (Van de Velde et al., 2019). D1 dopamine receptor (D1R) is a GPCR that activates cAMP/PKA signaling in neuron cells. BRD4 is recruited to dopamine-induced genes in response to cAMP/PKA signaling, and thereby increases the transcription of D1R-induced genes in striatal neurons (Jones-Tabah et al., 2022). Similarly, the activation of GPCR a1-adrenergic receptor (a1-AR) activated cAMP/PKA that increased BRD4 occupancy at promoters and super-enhancers of hypertrophic genes in cardiomyocytes (Martin et al., 2020).

Active P-TEFb is a crucial regulator for stimulating RNA polymerase II elongation. About half of P-TEFb associates with 7SK snRNA and HEXIM1 to form an inactive P-TEFb/HEXIM1/7SK snRNP complex. P-TEFb is hyperactivated that is mediated by PKA induced phosphorylation of HEXIM1 (Sun Y. et al., 2019) and cAMP induced nuclear translocation and condensate formation of CRTC2 (Mi et al., 2022) in ADPKD. Furthermore, P-TEFb is activated by BRD4. BRD4 interacted with P-TEFb, converted the 7SK/HEXIM1-bound P-TEFb into the BRD4-associated form. BRD4-associated P-TEFb functions as active form to promote transcription.

BRD4 was upregulated in *Pkd1* mutant mouse renal epithelial cell and tissues (Zhou et al., 2015). Inhibition of BRD4 with its inhibitor JQ1 delayed cyst growth and preserved kidney function in two PKD mouse models (*Pkd1* ^flox/flox^:*Pkhd1*-Cre and *Pkd1* ^nl/nl^). BRD4 has been identified as an upstream regulator of the expression of c-Myc in cystic renal epithelial cells. BRD4 promotes cystic renal epithelial cell proliferation through regulating c-Myc/p21 pathway. BRD4 has been reported to activate the transcription of target genes that are important for cell proliferation and apoptosis in a variety of cancers (Shorstova et al., 2021). Multiple clinical trials of BET inhibitors (BETi) in different cancers are ongoing (Shorstova et al., 2021). Apabetalone is a novel oral small molecule inhibitor of BRD4. Apabetalone reduced the incidence of major adverse cardiovascular events and showed favorable renal outcomes in patients with diabetic kidney disease and coronary artery disease in a phase 3 clinical trial (Kulikowski et al., 2018; Ray et al., 2020). Targeting BRD4 with apabetalone might be a potential therapeutic strategy for ADPKD.

## PKA, mitochondria and oxidative stress

Recent studies have identified functional and structural mitochondrial abnormalities in cyst lining cells of kidneys of patients with or mouse models of ADPKD. Mitochondrial oxidative phosphorylation is the main cellular source of ATP and reactive oxygen species. Reactive oxygen species can exert signaling roles or pathological effects depending on their levels. Mitochondria consist of an outer mitochondrial membrane, an intermembranous space, an inner mitochondrial membrane with folds called cristae, and a mitochondrial matrix.

Mitochondria contain three distinct cAMP-PKA signaling compartments, one tethered to outer membrane by different AKAPs, the second in the intermembrane space with PKA I tethered to the internal membrane by SKIP (sphingosine kinase-interacting protein), and the third confined to the mitochondrial matrix (Di Benedetto et al., 2021). cAMP and PKA signalling in the matrix and intermembrane space are mainly involved in the regulation of oxidative phosphorylation, whereas cAMP signaling in the outer membrane is involved in the regulation of mitochondrial dynamics, mitophagy, and apoptosis.

Oxidative phosphorylation consists of the transfer of two electrons from NADH (nicotinamide adenine dinucleotide) and FADH2 (flavin adenine dinucleotide) by enzymatic complexes I to IV (mitochondrial respiratory chain) within the inner mitochondrial membrane, coupled to proton extrusion at the level of complexes I, III, and IV into the intermembranous space, generation a proton gradient across the inner mitochondrial membrane, and phosphorylation of ADP to ATP by a process driven by the backflow of protons into the matrix involving ATP synthase (Zhao et al., 2019). Under physiological conditions, 0.2%–2% of the electrons leak out of complexes I, II and III and interact with oxygen to produce superoxide or hydrogen peroxide (Irazabal and Torres, 2020). PKA-dependent phosphorylation of the electron transport chain modulates oxidative phosphorylation (Figure 4) (Bouchez and Devin, 2019). PKA phosphorylates several subunits of complex I and is involved in the assembly and enzymatic activity of this complex. PKA also phosphorylates several subunits of complex IV increasing complex IV activity.

Structural mitochondrial abnormalities and reductions of mitochondrial copy number develop early in animal models of PKD and become more marked with disease progression (Cassina et al., 2020; Kahveci et al., 2020). Cells with a heterozygous *PKD1* mutation exhibit increased mitochondrial basal respiration and ATP production but decreased spared capacity with no difference in maximal mitochondrial respiration (Ishimoto et al., 2017). In contrast, cells with a homozygous *PKD1* mutation exhibit reduced basal respiration, ATP production, maximal respiration, spare capacity, and proton leakage. PKA activation may be responsible for the increased mitochondrial respiration in the cells with a heterozygous *PKD1* mutation since it was inhibited by the PKA inhibitor H-89. Increased nonmitochondrial respiration was found in both, cells with homozygous or heterozygous *PKD1* mutations. Oxidative stress may contribute to the accentuation of the structural and functional mitochondrial alterations with disease progression. Treatment with the mitochondrion-specific antioxidant MitoQ reduced mitochondrial superoxide production and proliferation of cyst-derived *PKD1* heterozygous cells. The mechanism(s) responsible for the early functional and structural mitochondrial alterations in PKD may only be partially understood. Cyclic AMP was found to be increased and calcium decreased in heterozygous *PKD1* cells. It was proposed that the increased cAMP-PKA activity was likely responsible for the early increase in mitochondrial respiration and superoxide generation, while reduced intracellular calcium acting on various regulators of PGC-1α expression (nitric oxide synthase, p38 MAPK and calcineurin) was responsible for its downregulation. Furthermore, downregulation of PGC-1α activates GSK3β and inhibits Nrf2 thus increasing the susceptibility of cystic kidneys to oxidative stress (St-Pierre et al., 2006).

Mitochondria form a dynamic network in the cytoplasm subject to biogenesis, fission, fusion and mitophagy (Mishra and Chan, 2016). Cyclic AMP and PKA signaling affects many of these processes. Mitochondrial biogenesis denotes the generation of new from existing mitochondria. It relies on nuclear transcription factors that regulate the transcription of most mitochondrial proteins, including those required for the transcription of the mitochondrial genome. PGC-1α regulates mitochondrial biogenesis by its ability to increase the expression and activity of NRF2 and subsequently of mitochondrial transcription factor A that promotes mitochondrial DNA replication (St-Pierre et al., 2006). PGC-1α also induces the expression of uncoupling protein-2, an inner mitochondrial membrane protein that dissipates the mitochondrial membrane potential, uncouples electron transport from ATP synthesis and reduces reactive oxygen species production (Vergnes et al., 2020). Downregulation of PGC-1α in PKD inhibits mitochondrial biogenesis.

Mitochondrial fission and fusion determine the size and number of mitochondria. Fission is regulated by dynamin-related protein 1 (Drp1) that recruits dynamin-2 to constriction points on the outer mitochondrial membrane (Di Benedetto et al., 2018). PKA-dependent phosphorylation of Drp1 retains it in the cytosol inhibiting fission. Inhibition of fission causes abnormal elongation of mitochondria. Nevertheless, both PKA dependent inhibition and promotion of fission have been reported (Chang and Blackstone, 2007; Cribbs and Strack, 2007; Cereghetti et al., 2008; Wikstrom et al., 2014; Di Benedetto et al., 2018). Inhibition of fission by PKA may favor fusion which is under the control of mitofusin 1 and 2 and optic atrophy 1. PKA also phosphorylates the mitochondrial membrane protein mitofilin, the clearance of damaged mitochondria (mitophagy) which is initiated by PTEN-induced putative kinase 1 (PINK1) and the E3 ubiquitin ligase Parkin (Akabane et al., 2016).

## PKA and inflammation

Activation of inflammatory pathways NFκB and STAT3, inhibition of anti-oxidative and anti-inflammatory Nrf2 signaling, and oxidative stress are associated with the upregulation of multiple inflammatory cytokines (TNF-α, IL-1, IL-6) and chemokines (MCP1) that promote cyst growth, interstitial inflammation and fibrosis. Cyclic AMP and PKA signaling may contribute to the development of interstitial inflammation and fibrosis by multiple mechanisms beyond the regulation of STAT3, NF- κB and Nrf2. Vasopressin has been shown to stimulate V2R/cAMP/PKA/ERK/YAP-mediated cell signaling in tubular epithelium, promote the expression and release of connective tissue growth factor (CCN2) and other YAP targets (PAI-1, AREG and MCP-1), and increase the number and activity of interstitial myofibroblasts and fibrosis in ADPKD kidneys (Figure 4) (Dwivedi et al., 2020). Renal tubule-specific YAP gene deletion and pharmacologic YAP inhibition using verteporfin attenuated the cystic disease in *Pkd1* knockout mice. Consistent with this observation, the administration of tolvaptan suppresses the excretion of MCP-1 in the urine (Grantham et al., 2017). This may account for the beneficial effect of tolvaptan at advanced stages of ADPKD (Torres et al., 2018b; Torres et al., 2021). Furthermore, cAMP has been shown in other tissues to induce monocyte recruitment in a manner dependent on PKA and MCP1/CCR2 signaling and to promote reprogramming of bone-marrow-derived macrophages to a M2 phenotype as seen by increased Arginase-1/CD206/Ym-1 expression and IL-10 levels (M2 markers (Polumuri et al., 2021)) through a PKA/C/EBPb/CREB dependent pathway (Negreiros-Lima et al., 2020).

## PKA and metabolic reprogramming

In the presence of oxygen, mammalian cells convert glucose to pyruvate (glycolysis) which enters the mitochondria to be completely oxidized through the mitochondrial tricarboxylic acid cycle where oxygen is the final acceptor in the electron transport chain (oxidative phosphorylation). Under anaerobic conditions (anaerobic glycolysis), stabilization of HIF-1α leads to the activation of glucose transporters, stimulation of enzymes promoting glycolysis (hexokinase, phosphofructokinase and pyruvate kinase), lactate dehydrogenase A, which enhances the flux of glucose through the glycolytic pathway while attenuating entry of pyruvate into the TCA cycle, and pyruvate dehydrogenase kinase, which inactivates the mitochondrial pyruvate dehydrogenase further inhibiting the entry of pyruvate into the tricarboxylic acid cycle and oxidative phosphorylation.

Rapidly proliferating cells repress oxidative metabolism and enhance glycolytic flux even under aerobic conditions (aerobic glycolysis (Vander Heiden et al., 2009; Lunt and Vander Heiden, 2011)). This metabolic reprogramming is required to support cell proliferation of rapidly growing tissues and is a feature of ADPKD, cancer and fetal development. It is characterized by inhibition of oxidative phosphorylation and fatty acid oxidation, stimulation of glycolysis and the pentose phosphate pathway, dependence on glutamine to provide intermediates to support the TCA cycle and lipid synthesis. The main goal of this metabolic reprogramming is the accumulation of glycolytic intermediates for biosynthetic purposes. Although glycolysis generates only 2 ATP molecules per molecule of glucose, as compared to 34 molecules of ATP per molecule of glucose generated by oxidative phosphorylation, the high glycolytic flux compensates for the lower efficiency.

mTOR and c-Myc are thought to play a central role in the metabolic reprogramming (Guertin and Sabatini, 2007; Duvel et al., 2010; Dejure and Eilers, 2017). In ADPKD, mTOR and c-Myc are consistently upregulated in PKD kidneys and repress oxidative metabolism, markedly enhance glycolytic flux (through upregulation of glucose transporters and the key glycolytic enzymes HK and PFK) and lactic acid production and export (through upregulation of LDH-A and monocarboxylic acid transporter 4, MCAT4 (Rowe et al., 2013; Podrini et al., 2020)). Nevertheless, mTOR and c-MYC are activated by PKA/MEK/ERK signaling and therefore activation of cAMP/PKA signaling may be a driver of metabolic reprogramming in some tissues. Furthermore, CREB has been implicated in metabolic reprogramming by targeting some critical glycolytic enzymes and PKA/CREB signaling has been found to drive metabolic reprogramming in some cancers (e.g., hepatocellular and prostatic carcinomas (Figure 4) (Moon et al., 2011; Sun et al., 2021)).

Because of their dependence on glycolysis and on glutamine PKD cells are particularly sensitive to glucose and glutamine deprivation. Inhibition of glycolysis with 2-deoxyglucose, a glucose analog that is not metabolized by cells, reduced cell proliferation in human PKD cells and kidney cyst growth in various murine and in a pig model (Chiaravalli et al., 2016; Riwanto et al., 2016; Lian et al., 2019). Furthermore, diet interventions such as caloric restriction, which attenuate PKD in several animal models may act through a similar mechanism (Kipp et al., 2016; Warner et al., 2016). Inhibition of glutaminase-1, which transforms glutamine into glutamate then catabolized into α-ketoglutarate, slowed cyst growth in *Aqp2*-Cre;*Pkd1* ^flx/flx^ but not in *Pkhd1*-Cre;*Pkd1* ^flx/flx^ mice (Flowers et al., 2018; Soomro et al., 2018).

## Discussion

Autosomal dominant polycystic kidney disease (ADPKD), with an estimated genetic prevalence between 1:400 and 1:1,000 individuals, is the third most common cause of end stage kidney disease after diabetes mellitus and hypertension (Bergmann et al., 2018). Over the last 3 decades there has been great progress in understanding its pathogenesis. A wealth of evidence supports that upregulation cAMP signaling promotes cystogenesis in ADPKD. Reduced polycystin function is thought to cause dysregulation of intracellular calcium, activation of adenylyl cyclases 5 and 6, inhibition of phosphodiesterase 1, and upregulation of cAMP and PKA signaling (Gattone et al., 2003; Torres et al., 2004; Wang X et al., 2005; Wang et al., 2008; Aihara et al., 2014). Upregulation of cAMP and PKA signaling may to a large extent be responsible for the disruption of tubulogenesis and initiation of cystogenesis (Song et al., 2003; Gallegos et al., 2012; Scholz et al., 2022) and for the progression of the cystic disease by stimulating fluid secretion (Sullivan et al., 1998; Grantham, 2003) and, in the setting of reduced intracellular calcium, epithelial cell proliferation (Yamaguchi et al., 2003; Yamaguchi et al., 2004; Yamaguchi et al., 2006). Previous studies have shown that constitutive activation of PKA not only causes a marked aggravation of PKD in mice with a hypomorphic *Pkd1* mutation but also induces a cystic phenotype in mice with a wild-type genetic background (Ye et al., 2017). Cyclic AMP analogs activating PKA, but not those activating Epac (exchange protein directly activated by cAMP), promote cystogenesis in *Pkd1* ^RC/RC^ metanephric organ cultures supporting the dominant role of cAMP/PKA signaling rather than Epac signaling in cystogenesis(Ye et al., 2017). Furthermore, constitutive inhibition of PKA attenuated PKD in *Pkd1* ^RC/RC^ mice (Wang et al., 2022). A recent study has shown that genetic inhibition of CREB suppresses cyst growth in ADPKD mouse models and that CREB orchestrates the expression of a cystogenesis associated transcriptome (Liu et al., 2021).

Interventions acting on G protein coupled receptors to inhibit of cAMP production have been effective in preclinical trials and have led to the approval of a vasopressin V2R antagonist (tolvaptan) for the treatment of rapidly progressive ADPKD (Torres et al., 2012; Torres et al., 2017). Their efficacy, however, is less than it can be achieved by the genetic elimination of circulating vasopressin in PCK rats, which causes massive polyuria and nearly completely abolishes the development of PKD but is not feasible in patients (Wang et al., 2008). Indeed the dosing of tolvaptan is limited by its powerful aquaretic effect. The proximal and central role of cAMP in the pathogenesis of ADPKD, the efficacy of tolvaptan and somatostatin analogs in preclinical and clinical trials, and the inability of these drugs to completely block renal cAMP production, provide a compelling rationale for exploring other targets in the cAMP signaling pathway. V2R antagonists affect PKA activity indirectly by lowering the production and tissue levels of cAMP. The tissue levels of cAMP can also be decreased by accelerating its degradation. Indeed, a PDE4 activator has been found to decrease kidney cAMP levels and attenuate the cystic disease to a degree comparable to that observed with tolvaptan with less polyuria in *Pkd1* ^RC/RC^ mice (Henderson et al., 2020). PKA activation in ADPKD can occur independently from cAMP. A novel PKA inhibitor also attenuated PKD in *Pkd1* ^RC/RC^ mice with only mild polyuria (Wang et al., 2022). CREB activation can also occur independently from PKA. A CREB inhibitor attenuated the cystic disease in ADPKD mouse models. Cyclic AMP/PKA/CREB upregulation affects many other downstream regulatory, signaling, and pathophysiologic pathways altered in ADPKD, as discussed in this review. Interventions targeting some of these downstream pathways may provide additive or synergistic value. In ADPKD, like in cancer and many other diseases, combinatory strategies with multiple drugs may be needed to achieve optimal results and tolerability. Currently the mechanisms by which cAMP promotes the development and progression of ADPKD are not completely understood and opportunities for targeting cAMP signaling in ADPKD are far from exhausted and remain feasible. A better understanding of cAMP/PKA/CREB/downstream signaling in ADPKD is likely lead to novel treatments that build on a strategy that has already been successful.
